# Coronary Intravascular Lithotripsy in Clinical Practice

**DOI:** 10.3390/jcm15176763

**Published:** 2026-08-31

**Authors:** Takashi Ashikaga, Toshihiro Nozato, Yasutoshi Nagata, Tetsumin Lee, Masakazu Kaneko, Toru Misawa, Masashi Nagase, Mao Matsuyama, Daigo Kachi, Maki Ohira, Kazuki Matsuda, Tomoki Horie

**Affiliations:** Department of Cardiology, Japanese Red Cross Musashino Hospital, 1-26-1 Kyonancho, Musashino 180-8610, Tokyo, Japan

**Keywords:** intravascular lithotripsy, optical coherence tomography, intravascular ultrasound, rotational atherectomy, orbital atherectomy, excimer laser coronary atherectomy

## Abstract

The presence of coronary artery calcification is associated with lower procedural success rates and worse long-term clinical outcomes. Atherectomy devices, such as rotational atherectomy (RA), orbital atherectomy (OA), and excimer laser coronary atherectomy (ELCA), have been used as preparation devices for severely calcified lesions. Intravascular lithotripsy (IVL) has recently been used to safely and selectively disrupt calcified coronary lesions. It produces acoustic shockwaves, which interact with the coronary calcium to create multiplanar fractures. These calcium fractures increase vessel compliance and result in a desirable minimal stent area or the ability to finish with a drug-coated balloon (DCB) alone strategy. The IVL has established its safety and efficacy for calcified lesions and its advantages over atherectomy devices include ease of use on a workhorse wire, ability to modify deep calcium, and less debris embolization causing slow-flow or no-reflow. A combined strategy of atherectomy devices followed by IVL may be efficient. We describe our experience with IVL and procedural technique in our catheterization laboratory.

## 1. Introduction

Coronary artery calcification negatively affects the outcomes of percutaneous coronary intervention (PCI) because of stent delivery failure and unsatisfactory stent apposition and expansion [1,2,3]. Stent underexpansion is associated with subsequent stent thrombosis and/or the need for target vessel revascularization [4]. Although atherectomy devices have been shown to improve luminal diameter and reduce the need for bailout stenting, vascular complications including distal embolization still remain [5]. Intravascular lithotripsy (IVL; Shockwave medical, Inc., Fremont, CA, USA) has emerged as a novel therapy for the treatment of vascular calcification [6]. The IVL system transforms electrical energy into mechanical energy during low-pressure balloon inflation. The technology of IVL does not rely on direct vascular tissue injury for plaque modification, but instead uses sonic waves, which travel from the balloon-based catheter to the surrounding tissue with the intention of safely and selectively breaking both superficial and deep calcium deposits with nominal soft tissue impairment, while improving vessel compliance. Here, we describe the tips and tricks of IVL in clinical practice.

## 2. IVL and Clinical Studies

The use of IVL in coronary artery disease (CAD) remains relatively recent, and the evidence is still limited to short-term outcomes only [7,8]. DISRUPT CAD I Trial demonstrated that the incidence of major adverse cardiac events (MACE) was low in patients with heavily calcified coronary lesions. They also showed that the major mechanism for calcium modification was the fractures on optical coherence tomography (OCT) findings [9]. DISRUPT CAD II Trial demonstrated that in-hospital MACE was low and calcium fractures by OCT were evident in 78.7% of lesions in patients with severe coronary calcified lesions [10]. DISRUPT CAD III Trial showed that freedom from MACE at 30 days and procedural success was 92.2% and 92.4% respectively and calcium fractures by OCT were evident in 67.4% of the lesions [11]. DISRUPT CAD IV Trial showed freedom from MACE at 30 days of 93.8% and 93.8% respectively and no complications occurred during the procedures [7]. In a meta-analysis, Sattar et al. demonstrated improvement in acute lumen gain and the success of stent delivery in severely calcified lesions [12]. Kaul et al. showed IVL was safer than rotational atherectomy (RA; Boston scientific, Natick, MA, USA) because IVL reduced the risk of atheromatous embolization. They also revealed that IVL yielded better results for acute lumen gain and residual stenosis compared to RA [13].

## 3. Mechanism of IVL

The IVL emitters employ electronic sparks to produce rapid expansion and collapse of vapor bubbles within the balloon, resulting in the production of acoustic pressure waves in the form of sonic pressure waves equivalent to approximately 50 atmospheres [14]. Acoustic pressure waves fracture the calcium after inducing contraction in it by creating a small peak of negative pressure. These acoustic pressure travels through soft tissues with minimal effect because of similar acoustic impedance parameters of fluid in the IVL balloon and soft tissue. This technology does not rely on direct vascular tissue injury for plaque modification but on sonic waves, which travel from the balloon-based catheter to the surrounding tissue with the intention of safely and selectively breaking into superficial and deep calcium deposits with minimal soft tissue impairment, while improving vessel compliance. As these waves are transmitted both circumferentially and transmurally in an unfocused manner, they affect both superficial and deep calcium. The acoustic energy delivery of IVL is circumferential and is not affected by wire bias, in contrast to other atherectomy technologies. The IVL is potentially the modality to modify thick and deep calcium without reducing calcium by atherectomy devices (Table 1) [15].

## 4. IVL Balloon System and Clinical Use

**Figure 1:**
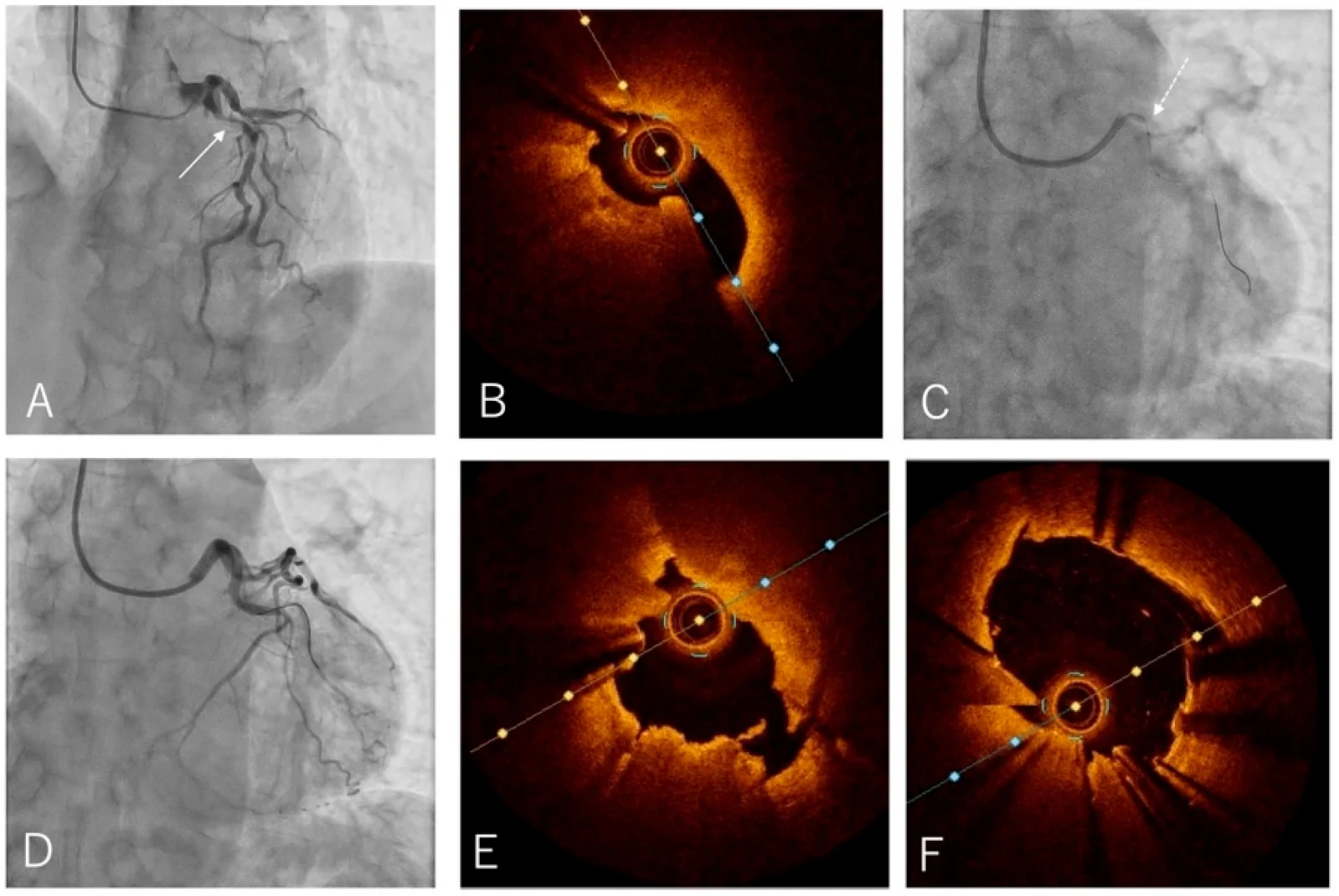
IVL balloon delivery with guide extension catheter for tortuous coronary artery. (**A**) Angiogram showed severe tortuous coronary artery. (**B**) OCT demonstrating eccentric calcium (5 to 12 o’clock). (**C**) A guide extension catheter (GEC) was needed to pass this lesion with an IVL balloon. (**D**,**E**) After using a 3.0 mm IVL balloon, lumen gain could be obtained in angiogram and OCT. (**F**) Final 3.5 mm DES was applied. White arrow indicates the lesion. White dotted arrow indicates the position of GEC.

The coronary IVL system consists of a single-use monorail catheter, a pulse generator, and a connector. The balloon size ranges from a diameter of 2.5 to 4 mm and a constant length of 12 mm, while its entry profile ranges from 0.042 to 0.046 inches at the level of two emitters mounted on a semi-compliant balloon. The current crossing profile is larger than that of traditional angioplasty balloon catheters because electrohydraulic lithotripsy emitters are integrated into the IVL catheter shaft. Then, it is sometimes difficult to deliver the IVL balloon particularly for severely tortuous lesions because of the reduced flexibility. In these cases, the semi-compliant balloon is sometimes required to promote the IVL balloon [16]. In addition, a guide extension catheter is sometimes also needed to deliver the IVL balloon to the culprit lesion (Figure 1).

**Figure 2:**
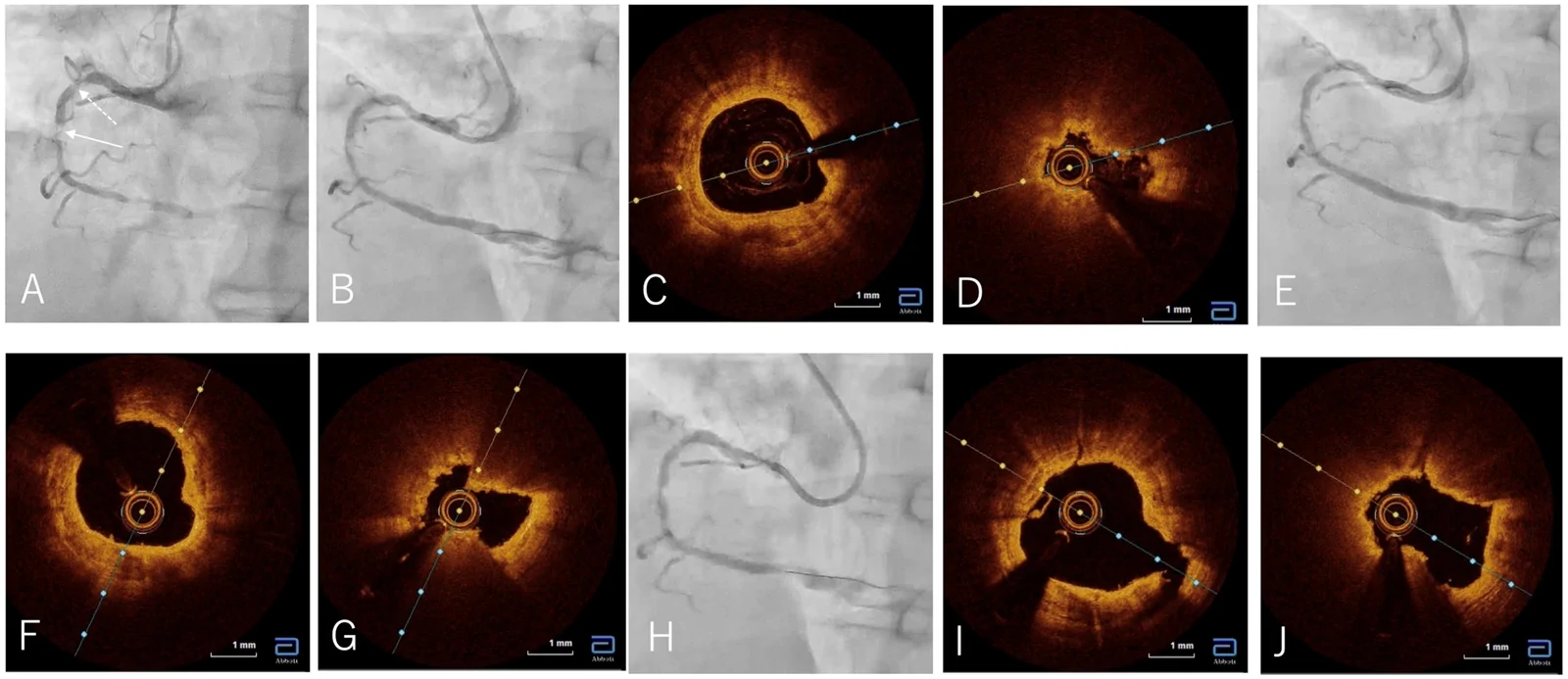
Adequate IVL balloon size is needed for calcium modification. (**A**) Angiogram. (**B**) Angiogram after 2.5 mm high-pressure balloon dilatation. (**C**,**D**) OCT showed no fractures and the lumen area was 5.45 mm^2^ and 1.98 mm^2^, respectively. (**E**) Angiogram after 2.5 mm IVL balloon. (**F**,**G**) After 2.5 mm IVL, OCT still showed no fractures, and the lumen area was 5.53 mm^2^ and 2.42 mm^2^, respectively. (**H**) Angiogram after 3.5 mm IVL balloon. (**I**,**J**) After 3.5 mm IVL, OCT showed fractures for superficial sheet at 4 o’clock (670 µm) and 12 o’clock (1040 µm) at the proximal site and a fracture for the eruptive calcified nodule at 12 o’clock at the distal site. Lumen area was 8.29 mm^2^ and 4.55 mm^2^, respectively. White dotted arrow indicates the proximal site. White arrow indicates the distal site.

Adequate balloon size is required for effective IVL, as sufficient contact of the expanded balloon with the luminal wall may be crucial for optimal lithotripsy energy dispersion [17] (Figure 2). In general, the ratio of the IVL balloon to the reference coronary diameter is recommended to be 1:1.

## 5. The Necessity of Additional Balloon Dilatation

**Figure 3:**
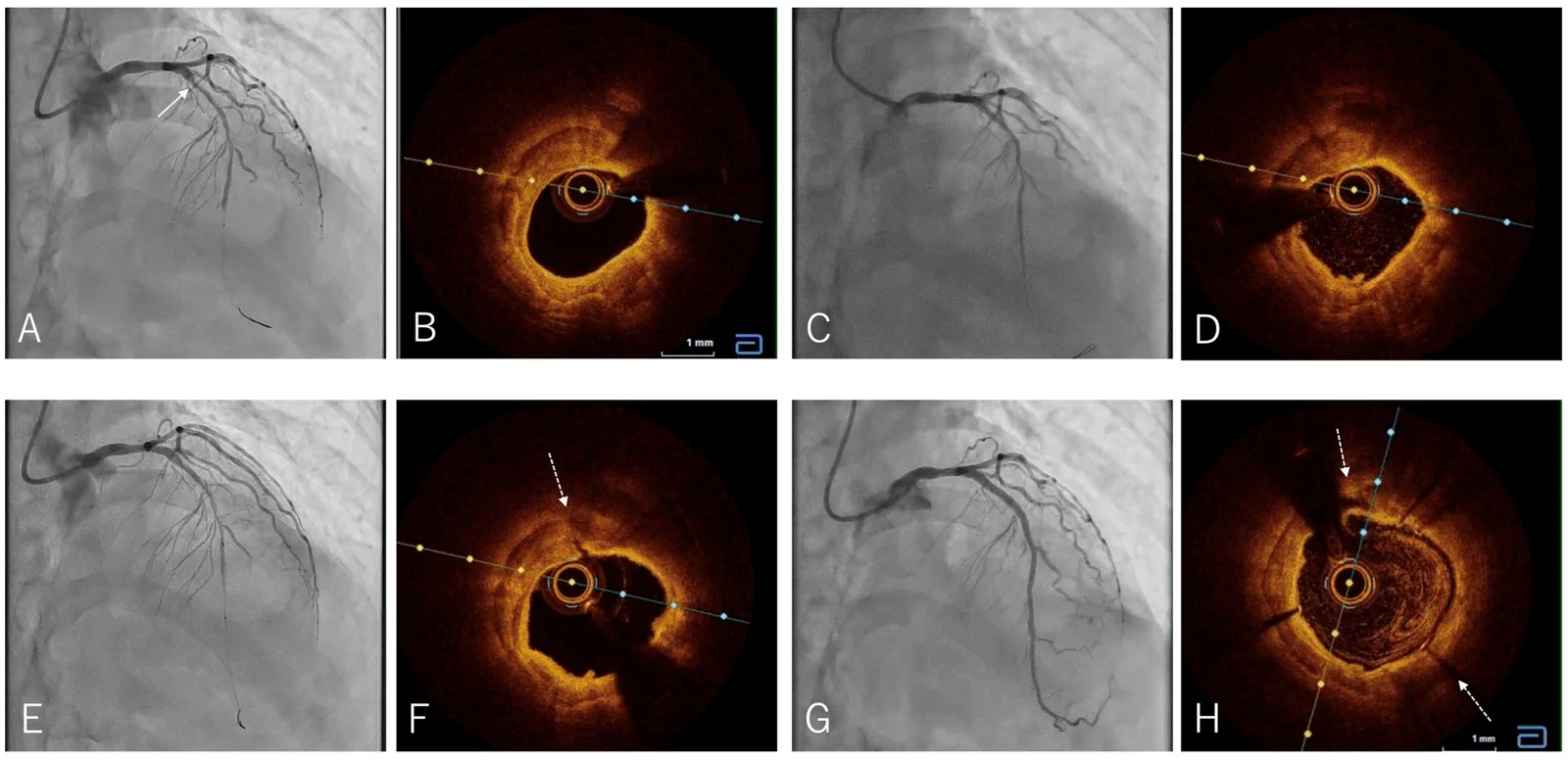
The existence of fractures after IVL followed by drug-eluting stent (DES). (**A**) Angiogram. (**B**) OCT showed a superficial calcified sheet. (**C**,**D**) Angiogram and OCT after 3.0 mm scoring balloon dilatation. No fracture was shown in OCT. (**E**,**F**) Angiogram and OCT after 3.0 mm IVL balloon. OCT showed fractures at 11 o’clock. (**G**,**H**) Angiogram and OCT after 3.25 mm DES deployment. OCT showed fractures at 11 and 4 o’clock beyond the stent strut. White arrow indicates the lesion. White dotted arrows indicate the fracture sites.

**Figure 4:**
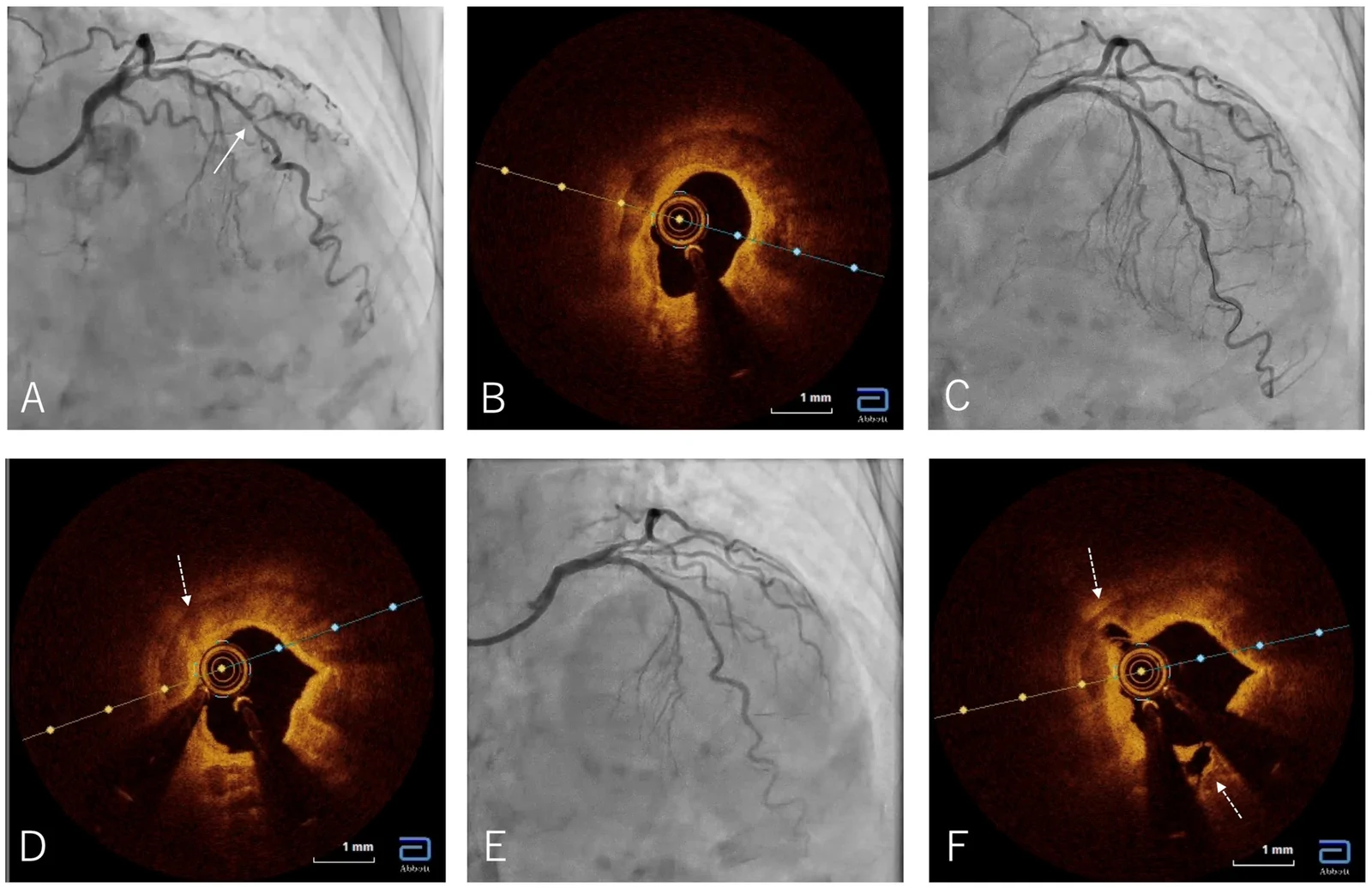
Microfracture after IVL followed by scoring balloon dilatation. (**A**,**B**) Angiogram and OCT. (**C**,**D**) Angiogram after 3.0 mm IVL balloon. A microfracture was seen at 10 o’clock in the OCT. (**E**,**F**) Angiogram and OCT after 3.0 mm scoring balloon dilatation. Fractures were evident at 10 and 6 o’clock. White arrow indicates the lesion. White dotted arrows indicate the fracture sites.

**Figure 5:**
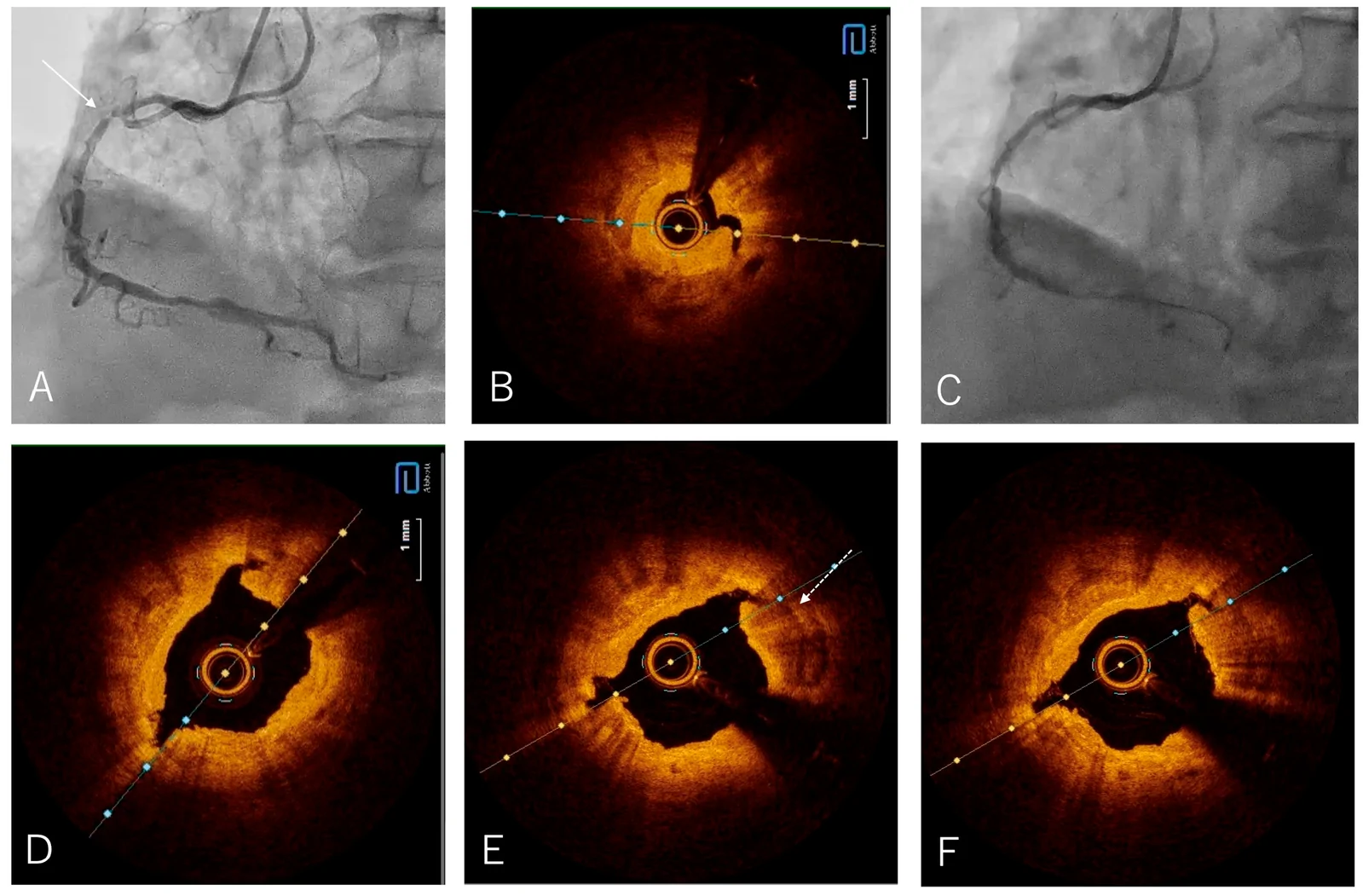
OCT stationary method demonstrating fractures during the cardiac cycle in IVL treatment. (**A**,**B**) Angiogram and OCT. OCT showed deep circumferential calcium. (**C**,**D**) Angiogram and OCT after 3.0 mm IVL. Fractures were evident at 12 and 7 o’clock in the OCT pullback. (**E**) A deep and circumferential fracture was evident at systolic phase in the OCT stationary method (1 to 3 o’clock). Superficial fractures were evident at 1 and 8 o’clock and the fracture at 8 o’clock was wider compared to that in the diastolic phase. (**F**) OCT at the diastolic phase using the OCT stationary method. White arrow indicates the lesion. White dotted arrows indicate the fracture sites.

The IVL-induced microfractures have been confirmed on histology and micro-CT. These fractures can then help achieve increased vessel compliance and stent area. Previous studies showed that the absence of fractures on imaging should not be taken into account as IVL failure because the final drug-eluting stent (DES) leads to adequate stent expansion. It is sometimes evident that fractures could be seen beyond the stent struts on optical coherence tomography (OCT) after DES deployment (Figure 3). Although the resolution of OCT is quite high, it is still not enough to confirm the fracture for IVL treatment. We can confirm the microfracture for IVL treatment because fractures can be seen in the same site after additional balloon dilatation (Figure 4) [18,19]. In addition, the existence of fractures could be demonstrated after the IVL treatment using OCT stationary analysis in spite of there being no fracture existence in OCT pullback analysis (Figure 5). Usual OCT analysis may underestimate microfractures with IVL treatment in clinical practice.

## 6. Effect of IVL for De Novo Calcified Lesion

**Figure 6:**
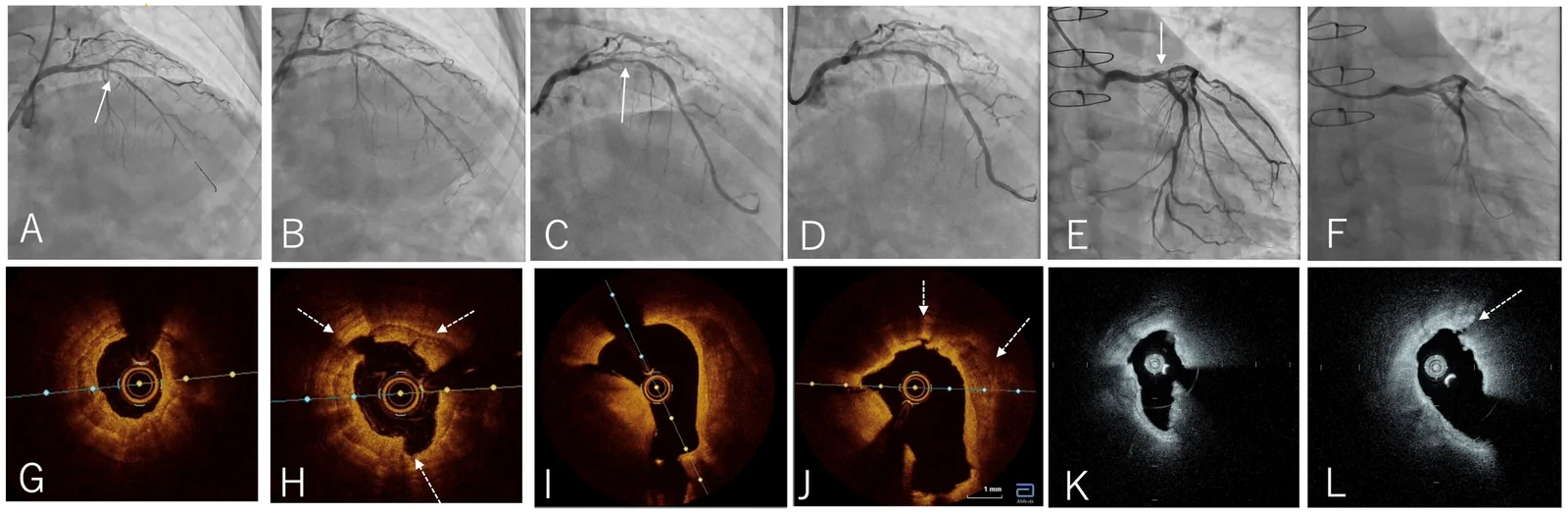
Modification effects of IVL. (**A**,**B**) Angiogram before and after IVL. (**C**,**D**) Angiogram before and after IVL (**E**,**F**) Angiogram before and after IVL. (**G**,**H**) Superficial calcified sheet treated with IVL. Lumen gain could be obtained and multiple fractures were evident at 1, 6 and 11 o’clock. (**I**,**J**) Non-eruptive calcified nodule treated by IVL. Lumen gain could be obtained and a fracture was evident at 12 o’clock and a deep and circumferential fracture was evident at 2 o’clock. A non-eruptive calcified nodule was compressed by IVL (7 to 9 o’clock). (**K**,**L**) Eruptive calcified nodule treated by IVL. Lumen gain could be obtained and a fracture was evident at 1 o’clock. White arrows indicate the lesions. White dotted arrows indicate the fractures.

The use of IVL is characterized into three types: superficial calcified sheet, non-eruptive calcified nodule, and eruptive calcified nodules (Figure 6). In clinical practice, IVL is also effective for eccentric and deep calcium modification in clinical practice.

### 6.1. Superficial Calcified Sheet

**Figure 7:**
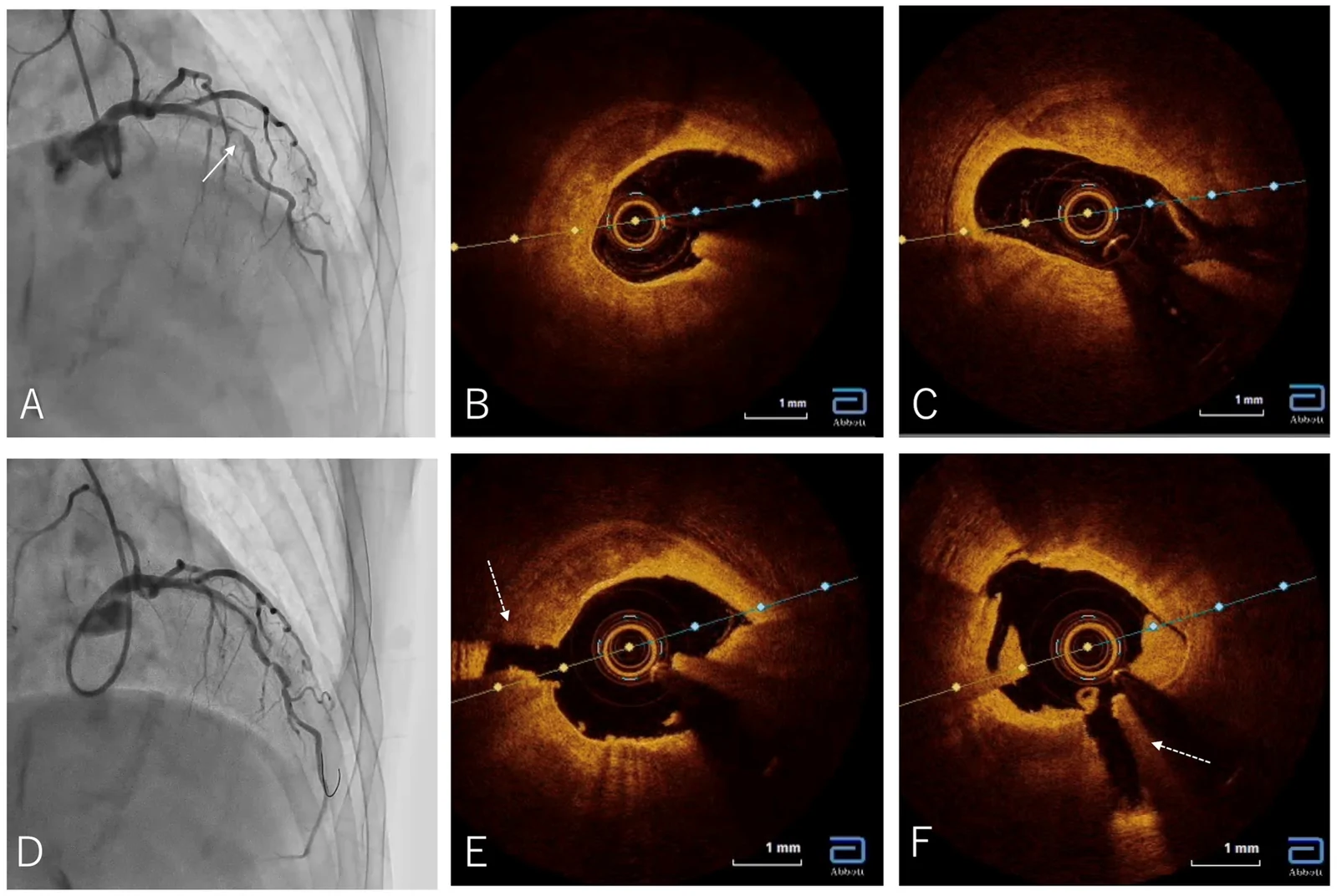
Superficial calcified sheet treated by IVL treatment. (**A**) Angiogram. (**B**,**C**) A superficial calcified sheet was seen in OCT. (**D**) Angiogram after 2.5 mm IVL. (**E**) Fracture was evident at 9 o’clock. Calcium thickness was 1100 µm. (**F**) Fracture was evident at 5 o’clock. Calcium thickness was 1650 µm. White arrow indicates the lesion. White dotted arrows indicate the fracture sites.

IVL uses acoustic pressure waves to interact with arterial calcification, producing micro and macro fractures in the calcified plaques allowing for increased vessel compliance and larger stent expansion. By fracturing the calcific layer, IVL improves vessel compliance without the need for aggressive high-pressure balloon dilatation prior to stent delivery. It thus reduces barotrauma to the vessel and consequently the chance of severe dissections. Debulking devices such as RA and orbital atherectomy (OA: Abbott Vascular, St. Paul, MN, USA) rely on localized debulking of superficial calcium, which subjects the target vessels to thermal injury and vascular complications. The primary mechanism of luminal gain following IVL treatment remains calcium fracture. These fractures are multi-planar, such as circumferential and longitudinal. Emori et al. showed that the thickness and angle of fractures within the superficial calcified sheet are greater compared to other type of balloon in clinical practice [20] (Figure 7).

### 6.2. Non-Eruptive Calcified Nodules

**Figure 8:**
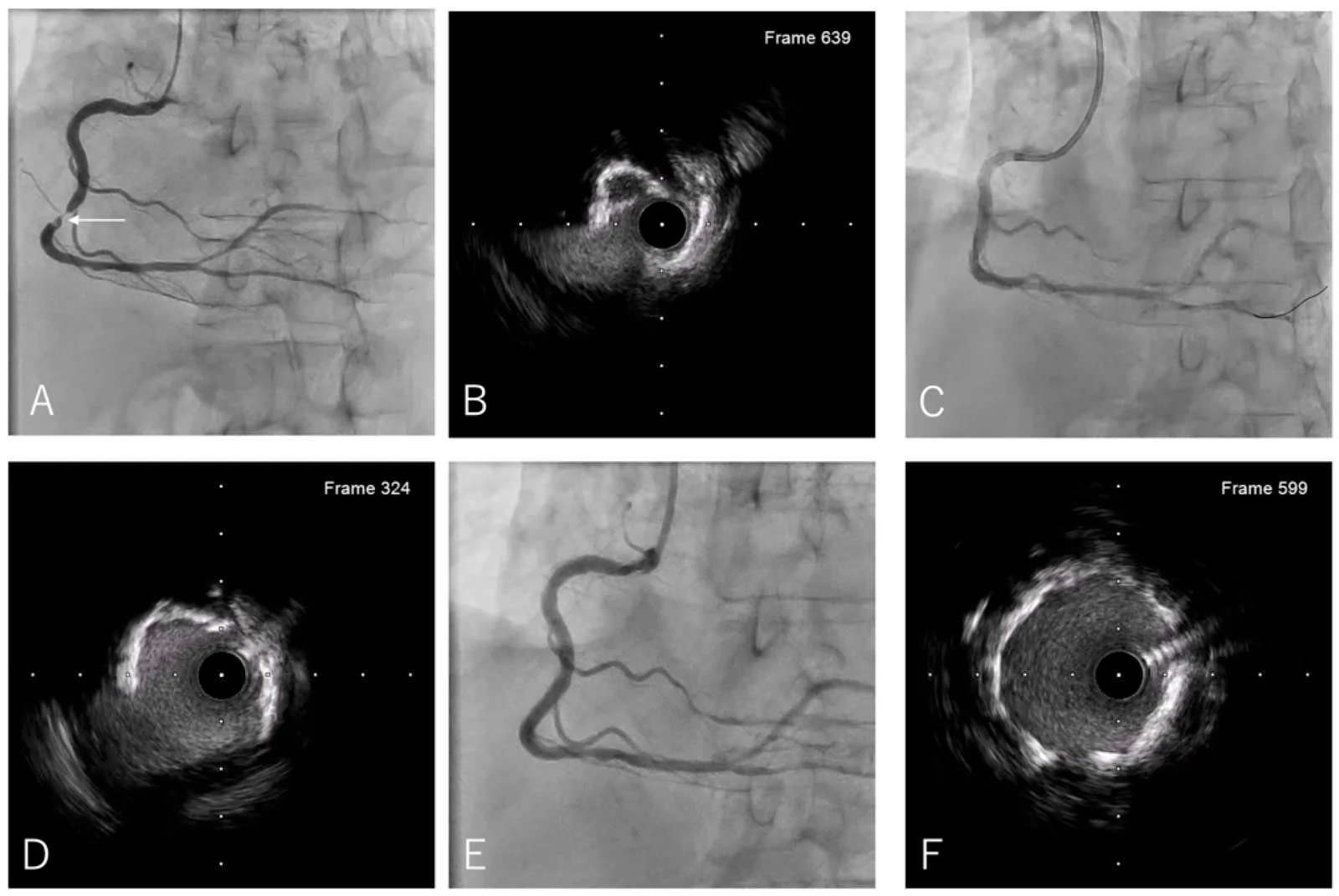
Calcified nodule treated with IVL. (**A**,**B**) Angiogram and IVUS. (**C**,**D**) Angiogram and IVUS after 3.5 mm IVL. Lumen gain can be seen on the angiogram and IVUS. (**E**,**F**) Angiogram and IVUS after 4.0 mm DES. Round-shaped dilatation can be seen on IVUS. White arrow indicates the lesion.

Under intravascular ultrasound (IVUS), a calcified nodule (CN) shows a convex luminal surface with a bright echo, bulging shape, irregular surface, acoustic shadowing, and an extensive calcium sheet at adjacent proximal and distal segments; however, it is difficult to differentiate between eruptive and non-eruptive CNs [21,22,23,24]. Even with or without eruptive CNs, IVL achieved lumen gain (Figure 8).

### 6.3. Eruptive Calcified Nodules

**Figure 9:**
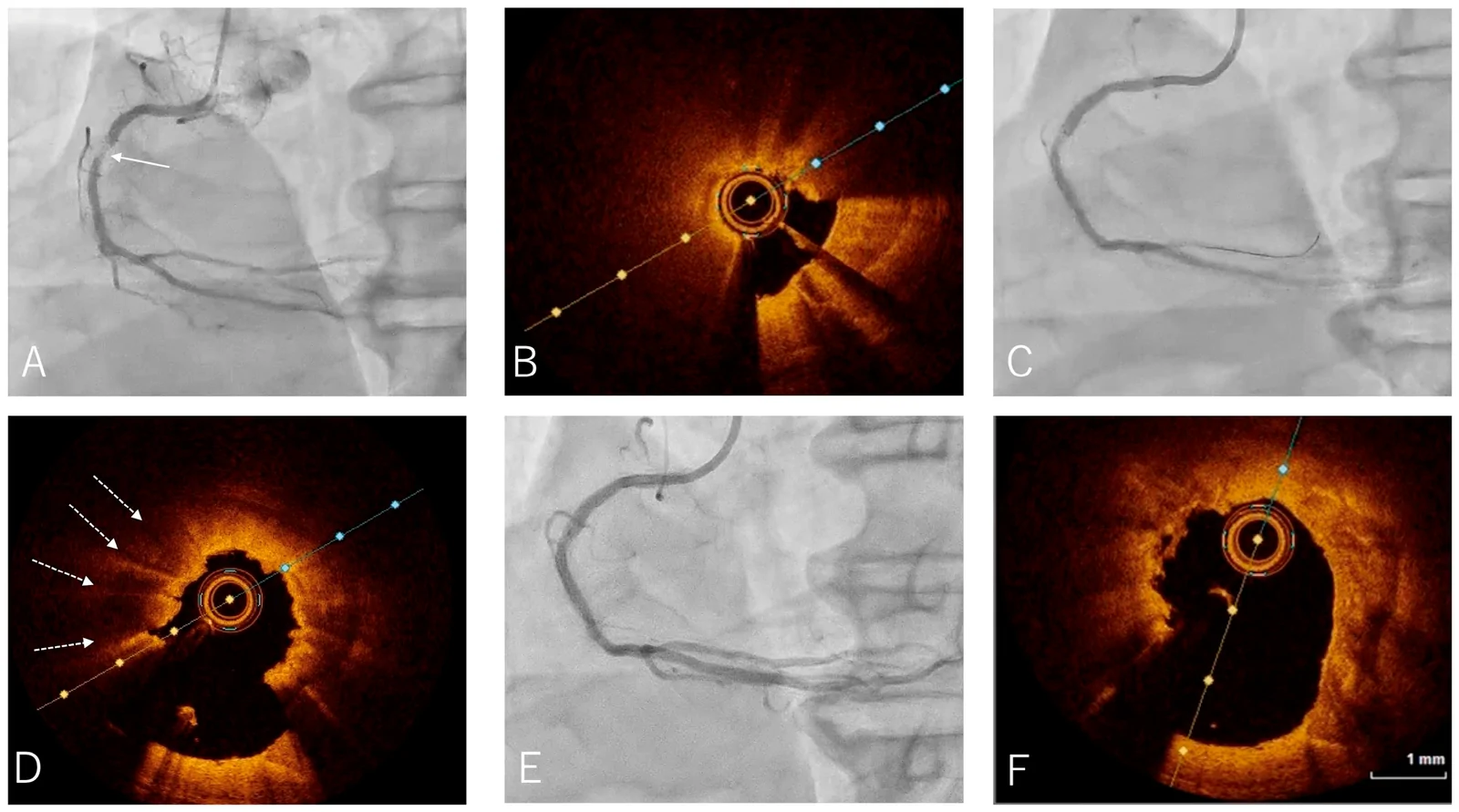
Eruptive calcified nodule treated by IVL. (**A**,**B**) Angiogram and OCT. (**C**,**D**) Angiogram and OCT after 3.0 mm IVL. Lumen gain was accomplished, and multiple fractures could be seen on OCT. (**E**,**F**) Angiogram and OCT after 3.0 mm scoring balloon dilatation. More lumen gain was accomplished. White arrow indicates the lesion. White dotted arrows indicate the microfractures.

**Figure 10:**
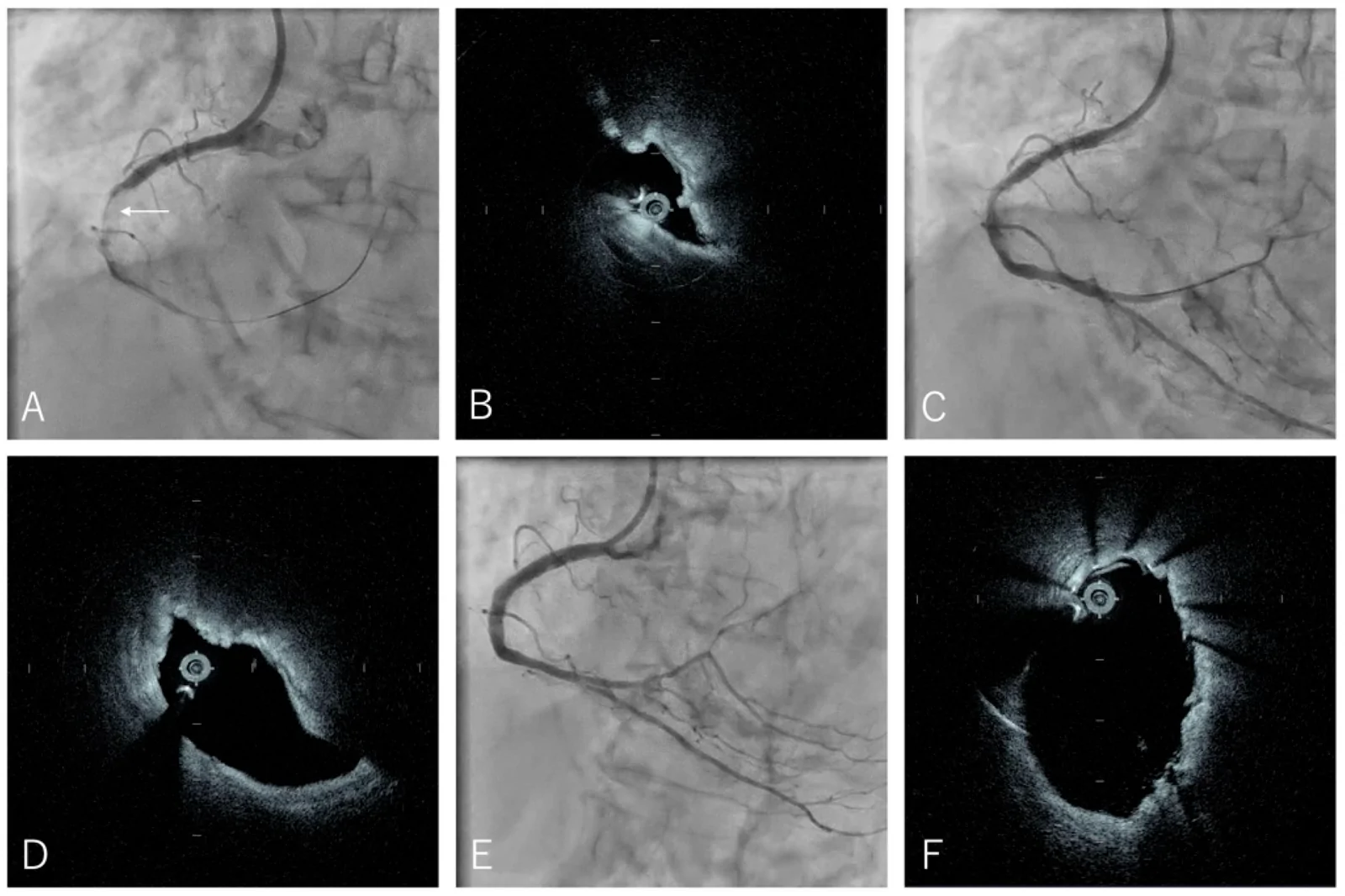
Eruptive calcified nodule treated by IVL in STEMI. (**A**,**B**) Angiogram and OCT demonstrating an eruptive calcified nodule, with the guidewire position far from the eruptive calcified nodule. (**C**,**D**) Angiogram and OCT after 3.0 mm IVL. The eruptive calcified nodule was compressed by IVL without vessel injury. (**E**,**F**) Angiogram and OCT after 3.5 mm DES. Lumen gain could be obtained with IVL followed by DES. White arrow indicates the lesion.

In OCT, eruptive CNs are indicated as a high backscattering protruding mass with an irregular surface covered by signal-rich bands adjacent to the luminal surface in more than two consecutive cross-sections, with fibrous cap disruption detected over a calcified plaque [25,26]. The prevalence of CNs has been reported in 22–40% of patients undergoing plaque modification [27,28]. A previous study showed that there were numerically higher rates of calcium fracture in CN and non-CN without complications. Although post-PCI stent expansion was similar in patients with and without CN lesions, CNs have been associated with worse outcomes and limited management options. Ali et al. examined the safety and efficacy of IVL in CNs from disrupt I–IV trials and found that it was a highly effective modality with similar procedural outcomes when compared to non-CN lesions in terms of residual area stenosis, stent expansion or acute gain [29] (Figure 9). Since many cases with CNs are eccentric, the position of the guidewire is very important for the use of debulking devices. Hence, IVL treatment is easy to use because IVL does not rely on the guidewire bias [30] (Figure 10).

### 6.4. Eccentric Calcium

**Figure 11:**
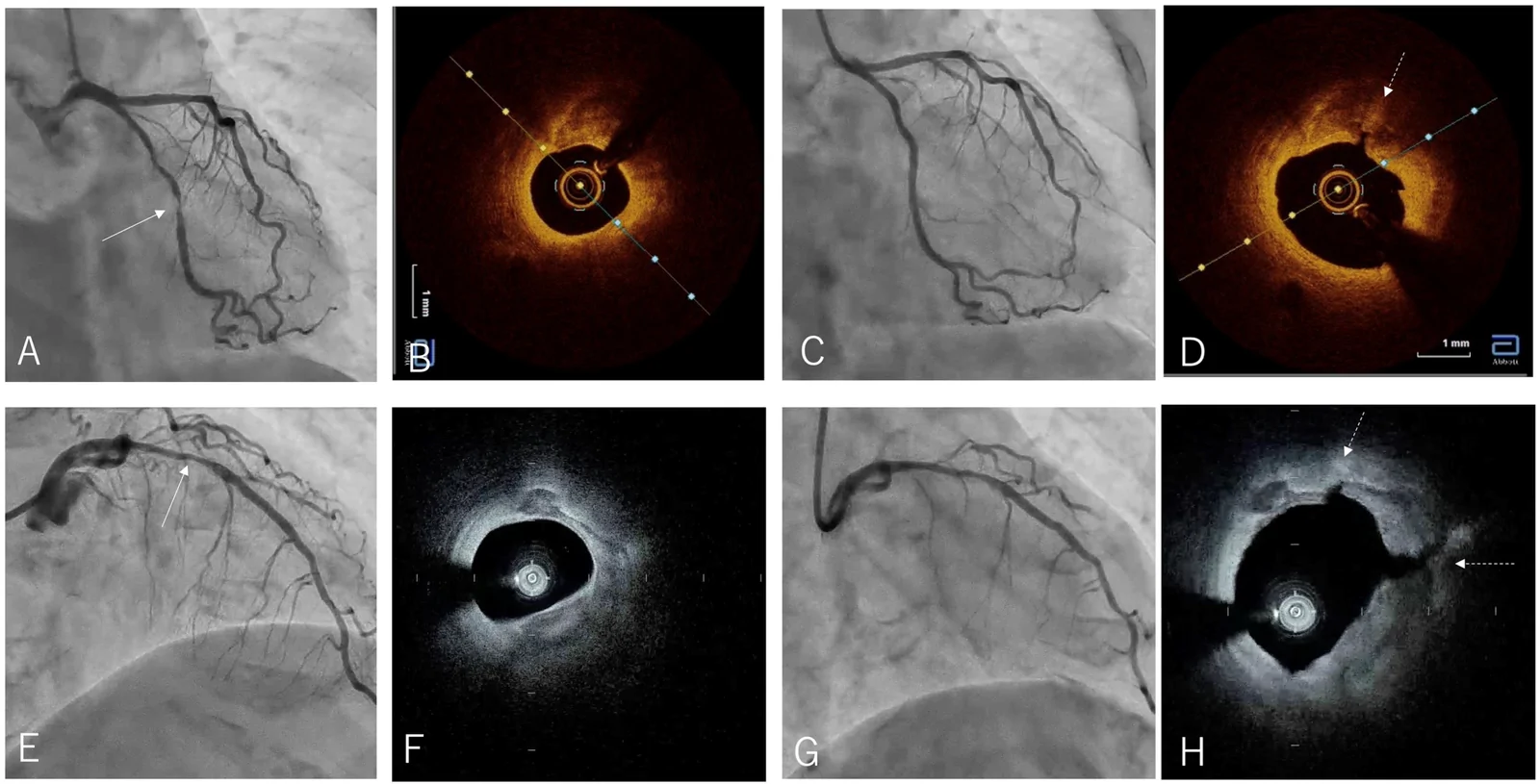
Two cases of eccentric calcium treated by IVL. Top is the first case. Bottom is the second case. (**A**,**B**) Angiogram and OCT. (**C**,**D**) Angiogram and OCT after 2.5 mm IVL. Lumen gain with fracture at 12 o’clock was seen in the OCT. (**E**,**F**) Angiogram and OCT. (**G**,**H**) Angiogram and OCT after 3.0 mm IVL. Lumen gain with multiple fractures were seen at 12 and 3 o’clock in the OCT. Circumferential fractures were seen from 12 to 4 o’clock. White arrows indicate the lesions. White dotted arrows indicate the fractures.

Eccentric calcium present in target lesions often creates additional difficulty for PCI, as the use of calcium modification techniques may be limited. Non-compliant, cutting and scoring balloons may direct force away from the eccentric calcified lesion and can lead to dissection or vessel perforation due to expansion towards the more compliant non-calcified segment. Circumferential calcium is not necessary for sonic waves to modify with IVL, and fractures could be made by IVL treatment (Figure 11). Final DES and DCB strategy may be feasible and acceptable if fractures within superficial calcium can be made by IVL treatment.

### 6.5. Deep Calcium

**Figure 12:**
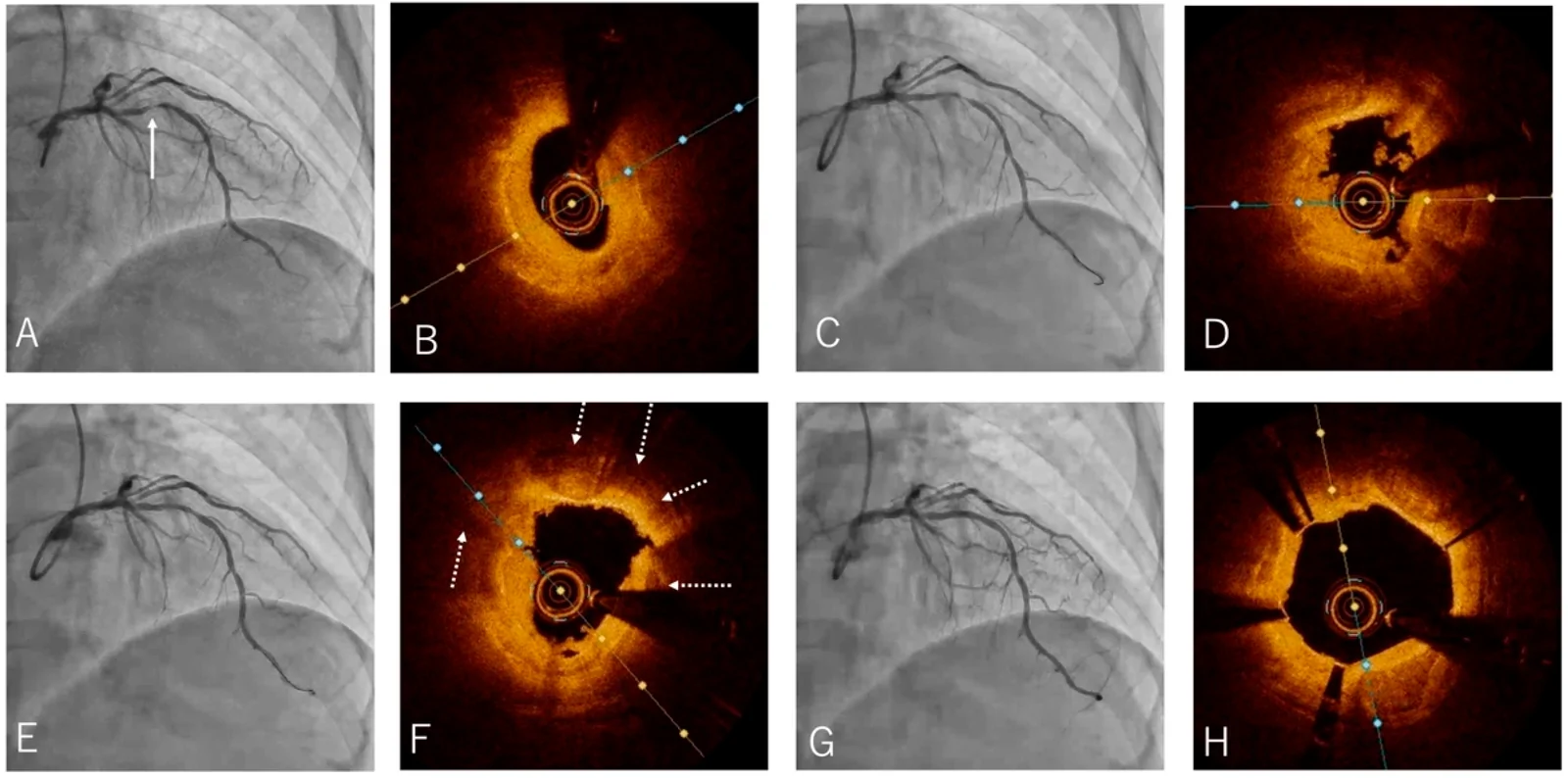
Deep calcium treated by IVL. (**A**,**B**) Angiogram and OCT. (**C**,**D**) Angiogram and OCT after 2.5 mm scoring balloon dilatation. No fracture was seen on OCT. (**E**,**F**) Angiogram and OCT after 2.5 mm IVL. Multiple fractures were evident in the deep calcium. (**G**,**H**) Angiogram and OCT after 3.0 mm DES. Adequate stent expansion was obtained after DES. White arrow indicates the lesion. White dotted arrows indicate the fractures in deep calcium.

It is quite difficult to make fractures in deep calcium with debulking devices and cutting/scoring balloon dilatation. The modification of deep calcium may be effective in increasing the target vessel compliance. In addition, by fracturing deep calcium, lumen gain and adequate stent expansion could be obtained (Figure 12).

## 7. Effect of IVL for Calcified In-Stent Restenosis (ISR)

ISR is divided into two groups in view of the existence of calcified lesions. Calcified ISR were divided into three groups in view of OCT findings such as stent underexpansion, calcified neoatherosclerosis and ISR-related CNs [31,32].

### 7.1. Stent Underexpansion

**Figure 13:**
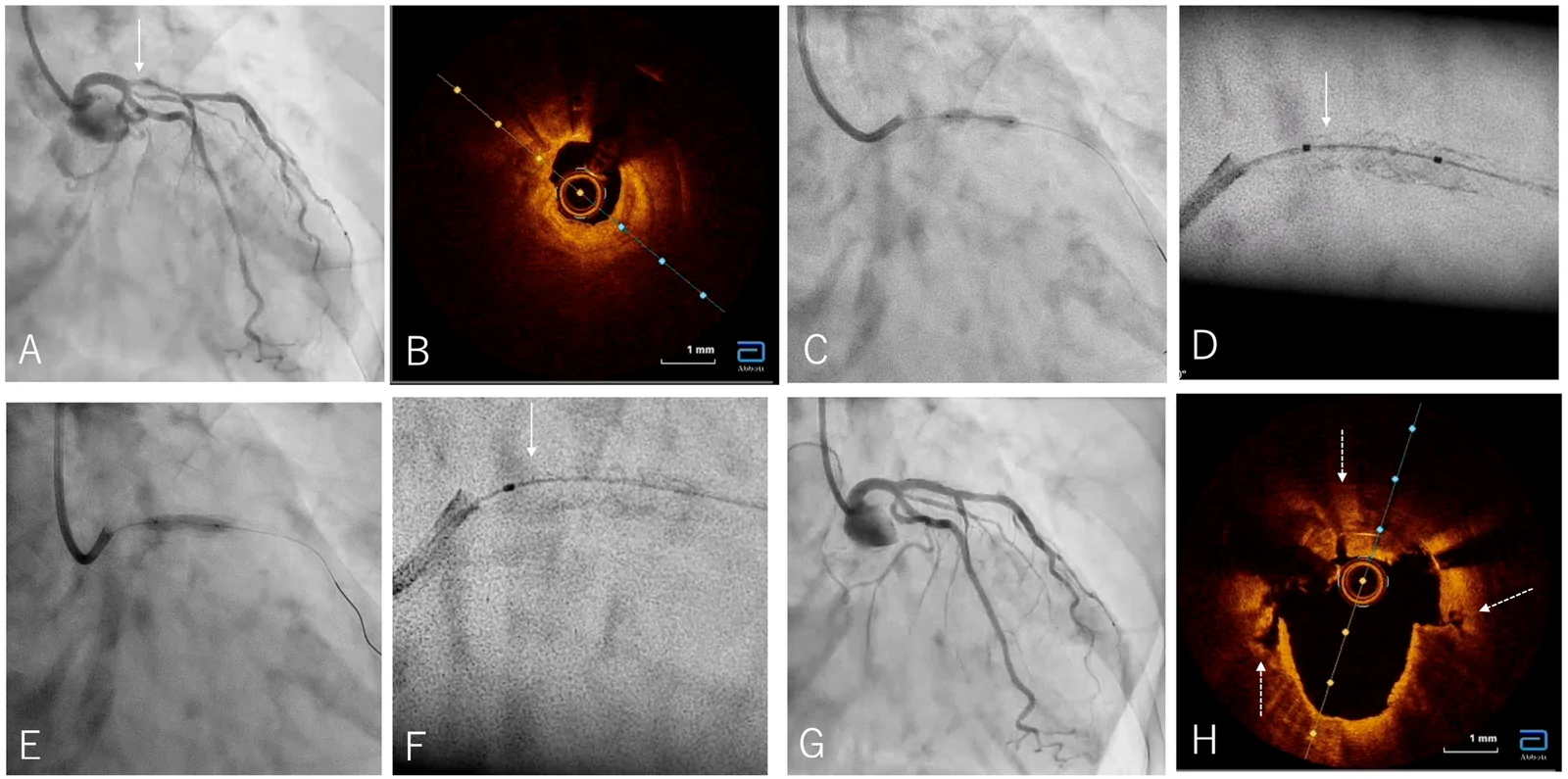
Stent under expansion with calcified neoatherosclerosis treated by IVL. (**A**,**B**) Angiogram and OCT. The stent was deployed 19 years ago. OCT showed that thick calcium was located within and beyond the stent strut. Lumen area and stent area were 1.49 mm^2^ and 5.99 mm^2^, respectively. (**C**) Angiogram demonstrating dog bone effect by 3.0 mm scoring balloon dilatation at 24 atm. (**D**,**E**) Angiogram showed stent underexpansion. IVL emitter was placed at the stent underexpansion site. 3.0 mm IVL at 4 atm was dilated. (**F**) Angiogram showed that the stent itself was well dilated. (**G**,**H**) Angiogram and OCT after 3.0 mm IVL. Lumen area and stent area were 5.13 mm^2^ and 8.08 mm^2^, respectively. respectively. Multiple fractures were made within and beyond the stent struts. White arrows indicate the lesion. White dotted arrows indicate the fractures in thick calcium within and beyond the stent strut.

Undilatable lesions have been treated with cutting/scoring balloon and/or atherectomy devices. However, debulking devices have some risks and cutting/scoring balloons are sometimes not effective for stent underexpansion due to thick calcium. Circumferential sonic waves of IVL have the advantages of extending beyond stent layers and fracutring deeper calcium deposits. Several case reports have supported the use of the IVL technology for optimizing stent expansion without complications [33,34,35,36,37] (Figure 13). However, the use of IVL for underexpanded stents is still an off-label indication, as there is a concern of IVL -induced polymer disruption of the stent. An ex vivo study of an everolimus-eluting fluoropolymer-coated drug-eluting stent demonstrated microscopic cracks, tears and detachment of the fluoropolymer coating after delivering 80 shocks with an IVL balloon. However, the overall integrity of the fluoropolymer remained preserved and the degree of disruption was not enough to be significant enough to interfere with antiproliferative drug delivery [38].

### 7.2. Calcified Neoatherosclerosis

**Figure 14:**
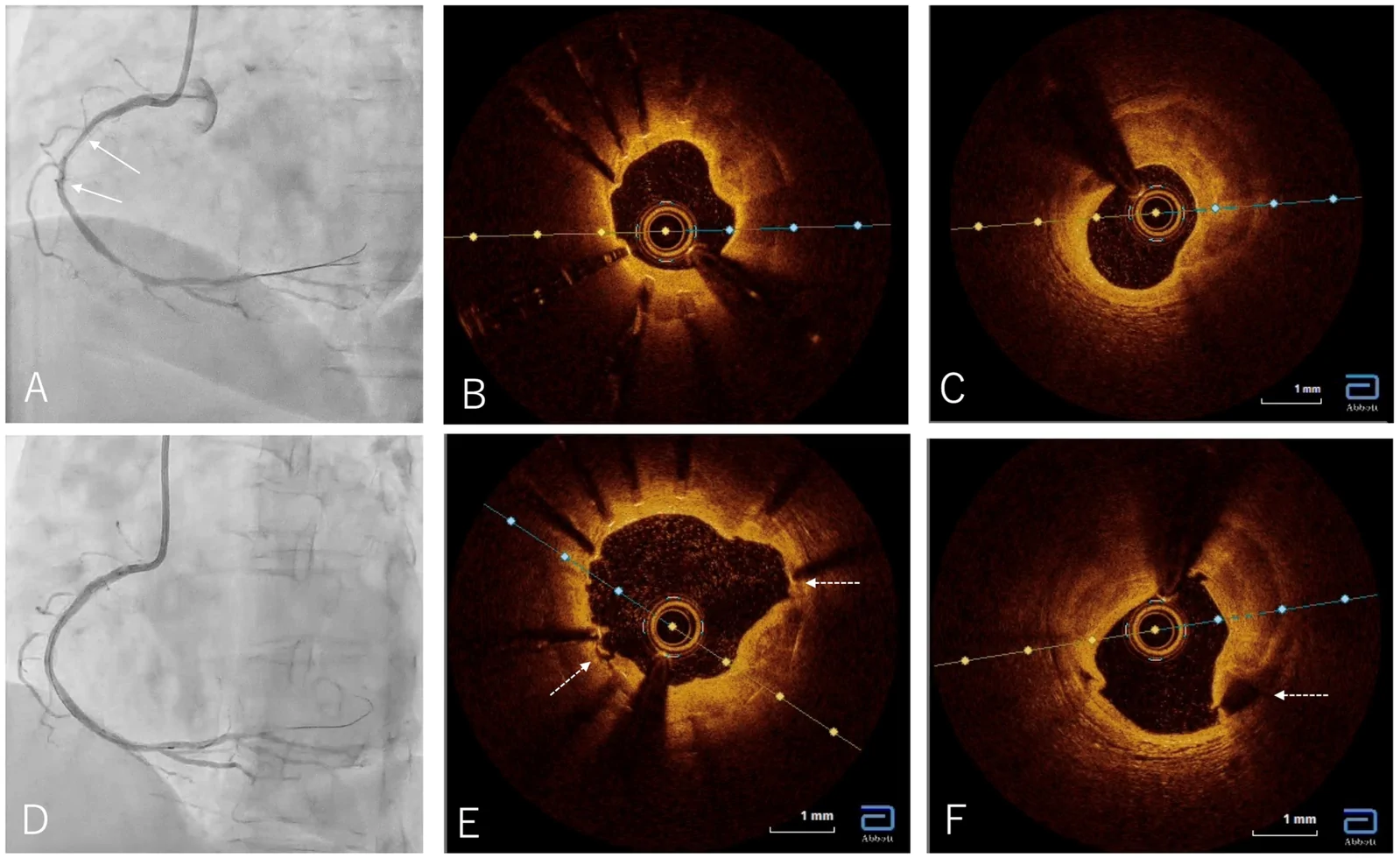
Stent underexpansion with calcified neoatherosclerosis and eccentric calcium treated by IVL. (**A**–**C**) Angiogram and OCT. OCT demonstrating calcified neoatherosclerosis with stent underexpansion and de novo superficial calcified sheet. OCT showed that the lumen area and stent area were 2.18 mm^2^ and 5.04 mm^2^, respectively. (**D**–**F**) Angiogram and OCT after 2.5 mm IVL. OCT showed that lumen area and stent area were 6.49 mm^2^ and 9.81 mm^2^, respectively. Multiple fractures could be seen in both the ISR and the de novo calcified lesion. White arrow indicates the lesion. White dotted arrows indicate fractures.

Even now, debulking devices and balloon-based therapies are still the main therapies for ISR due to calcific neoatherosclerosis [39]. Although IVL has been tested for this lesion, the IVL balloon may not advance into the lesion if the lesion is extremely severe or there is tortuosity at the proximal part of the artery. Since both de novo calcified lesions and calcified neoatherosclerosis coexist, IVL could modify and dilate both calcified lesions well, including the stent itself (Figure 14).

### 7.3. ISR-Related Calcified Nodules

**Figure 15:**
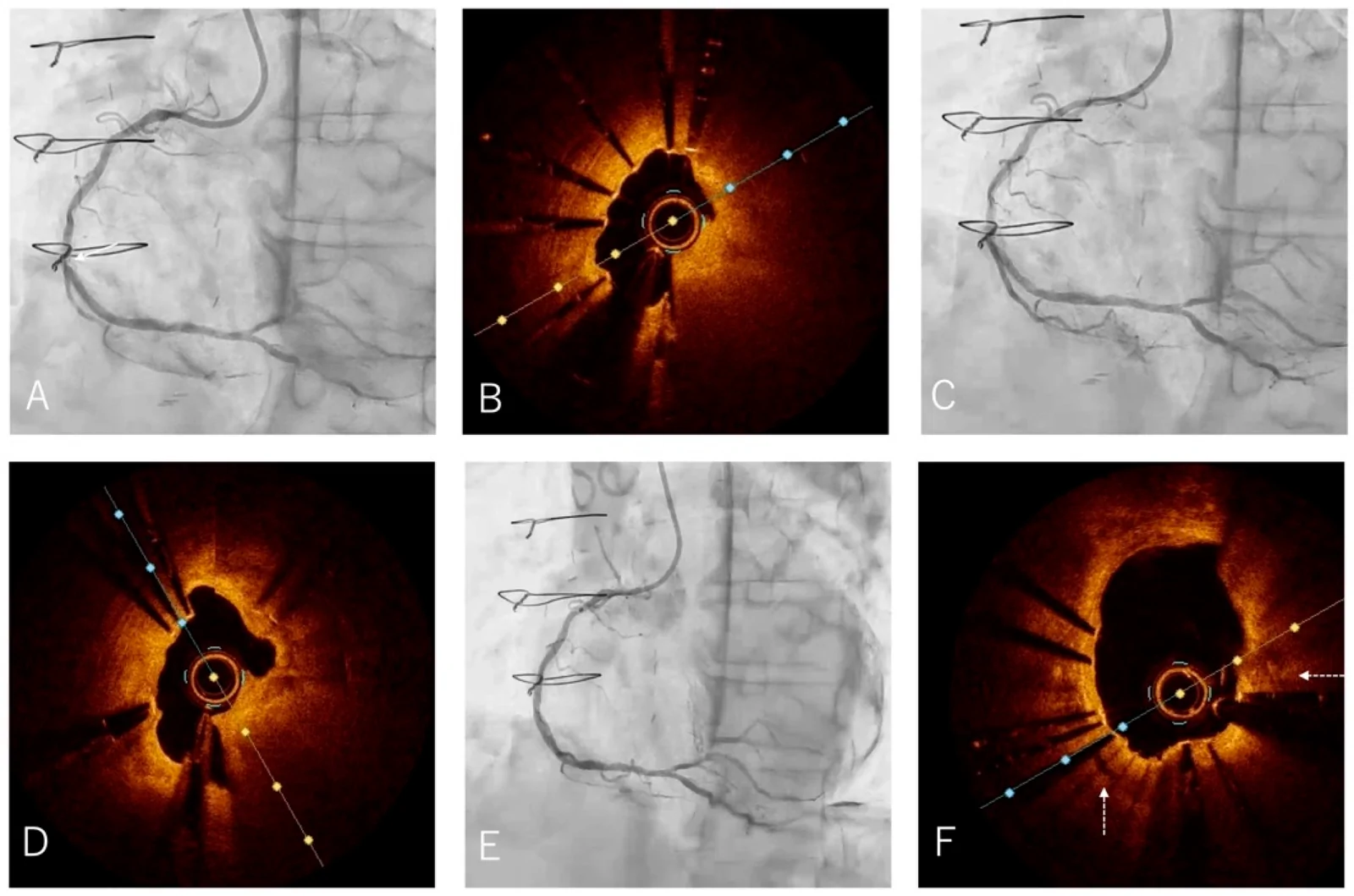
ISR-related calcified nodule treated by RA followed by IVL. (**A**,**B**) Angiogram and OCT. OCT showed an ISR-related calcified nodule. (**C**,**D**) Angiogram and OCT after 1.5 mm RA. Calcified nodule reduction was seen on OCT. (**E**,**F**) Angiogram and OCT after 3.0 mm IVL. Lumen gain and circumferential fractures were seen on OCT. White arrow indicates the lesion. White dotted arrows indicate the fractures.

ISR-related CNs within the stent could develop in around 10% of all ISR lesions, particularly within stents deployed in severely calcified lesions, and such CNs cause mechanical stress, resulting in the reprotrusion of CNs with or without visible stent fracture, frequently causing recurrent thrombotic events. ISR-related CNs have also been shown to be associated with worse outcomes [40,41]. Debulking devices followed by IVL may be a therapeutic option (Figure 15).

## 8. IVL for Specific Angiographic Situations

### 8.1. Bifurcation Lesions

**Figure 16:**
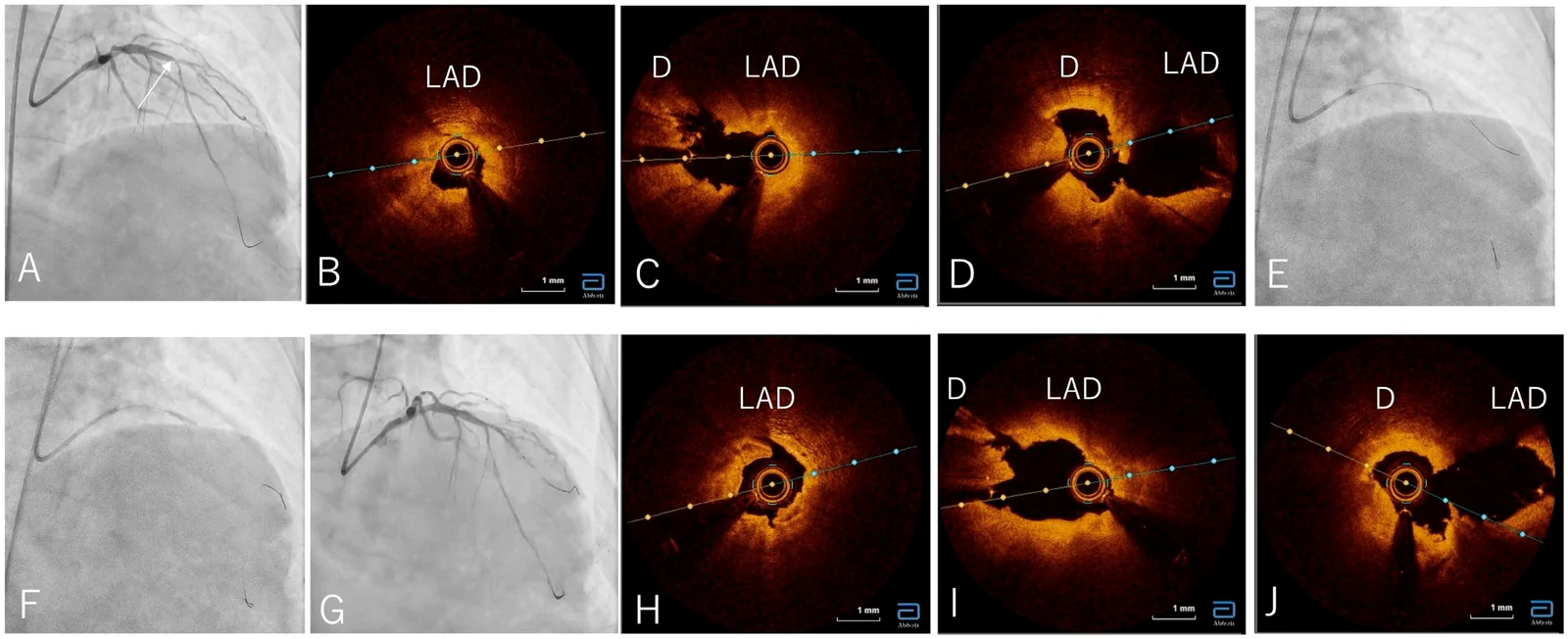
IVL for bifurcation lesion. (**A**–**D**) Angiogram and OCT. OCT showed severe calcium in both the left anterior descending artery (LAD) and the diagonal branch (**D**). (**E**) 2.5 mm IVL for LAD. (**F**) 2.5 mm IVL for D. (**G**–**J**) Angiogram and OCT after sequential 2.5 mm IVL. Multiple fractures were seen in the proximal site; however, no fracture was seen in the bifurcation area. White arrow indicates the lesion. Abbreviations: LAD; left anterior descending artery, D; diagonal branch.

**Figure 17:**
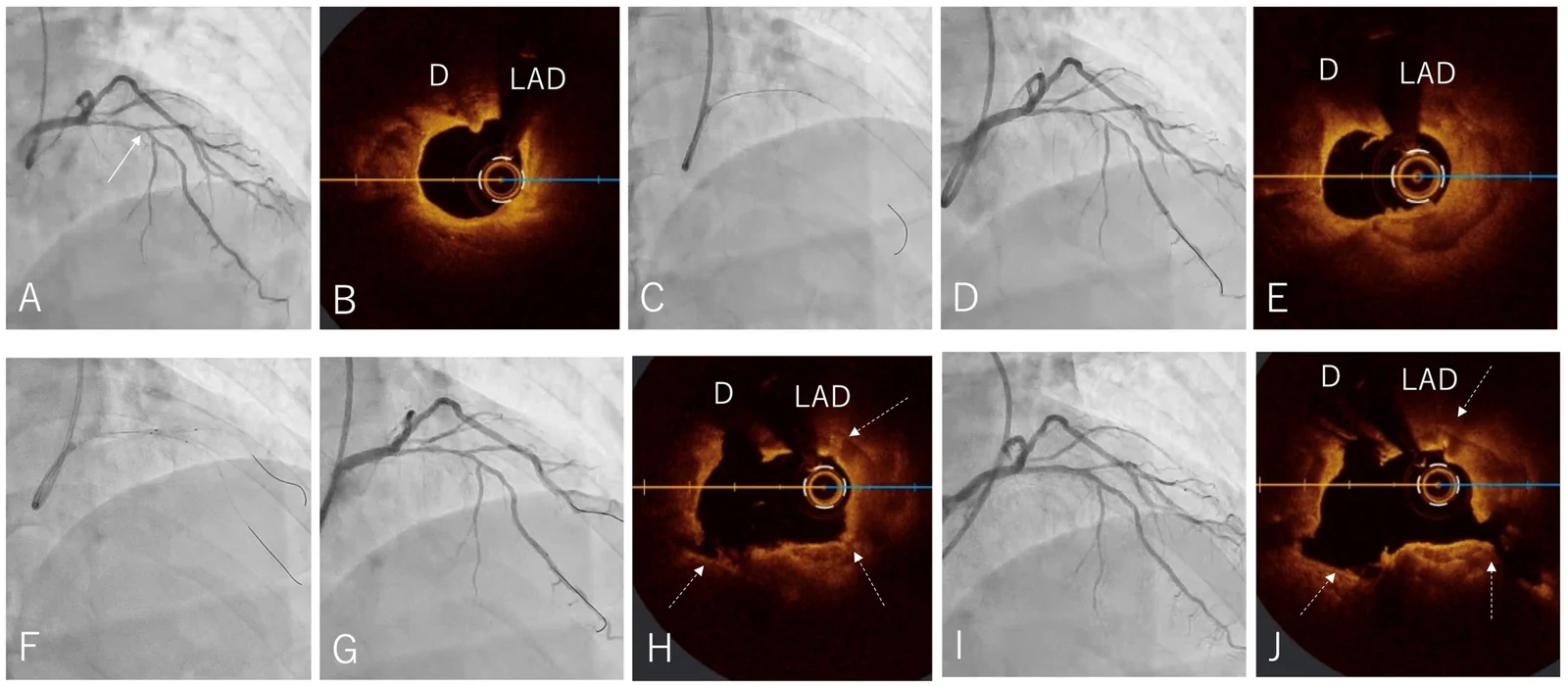
OA followed by IVL for bifurcation lesion. (**A**,**B**) Angiogram and OCT. Thickness of calcium was 1500 µm at 3 o’clock. (**C**–**E**) Angiogram and OCT after 120,000 rpm OA. Thickness of the calcium was 1160 µm at 3 o’clock. (**F**–**H**) Kissing balloon inflation with a 2.5 mm IVL balloon (LAD) and a 1.5 mm regular balloon (Diagonal branch). Multiple fractures were seen in LAD (1 and 4 o’clock) and fracture in diagonal branch (7 o’clock). (**I**,**J**) Angiogram and OCT after additional scoring balloon. Fractures were evident at 1, 4, and 7 o’clock, and lumen gain was obtained. White arrow indicates the lesion. White dotted arrows indicate the fractures. Abbreviations: LAD; left anterior descending artery, D; diagonal branch.

Coronary bifurcation lesions account for approximately 15–20% of all procedures and are associated with a high risk of procedural complications and long-term MACE compared to non-bifurcation lesions [42]. The presence of extensive calcification within bifurcations further increases procedural complexity by impeding balloon and stent delivery, limiting adequate preparation, and raising the risk of side branch occlusion [43,44]. Modification devices, such as RA and OA, might optimize the results [45]. In the PREPARE-CALC study, side branch (SB) compromise was observed less with RA compared to cutting and scoring balloons [46]. Chambers et al. demonstrated that either OA or RA documented similar low 30-day MACE rates among patients with bifurcation as compared with non-bifurcation lesions. In addition, they also showed that the procedure and fluoroscopy time were significantly shorter in OA compared with RA [47]. In addition, OA in bifurcation lesions was associated with low MACE rates similar to those observed for non-bifurcation lesions in a large, multicenter analysis of pooled data [48]. However, both RA and OA may pose some technical challenges because of the need for single wire use, impeding the protection of SB. In addition, RA carries an increased risk of SB perforation and contrast-induced nephropathy in bifurcation lesions [49]. The priority of IVL is that protective wires can be maintained throughout the procedure [50]. In addition, IVL offers several advantages over atherectomy in balloon-crossable lesions and minimal thermal injury without causing debris embolization including ease of use with a standard workhorse wire, the absence of guidewire bias, and the ability to modify and fracture deep calcium in situ [51,52]. On the contrary, a previous report showed that IVL leads to decreased luminal gain in bifurcation lesion [53]. However, they had not confirmed the true mechanism of acute luminal loss, which may be due to plaque shift and/or carina shift. In clinical practice, the balloon size of IVL is important because smaller IVL balloons are difficult to make fractures for both the main branch (MB) and SB. (Figure 16). In our experience, the kissing balloon technique using the IVL balloon and a regular balloon was effective for acute lumen gain with evident fractures for both the MB and SB (Figure 17). Since both the IVL balloon and the regular balloon can contact the vessel wall, the acoustic pressure caused by the IVL balloon may lead to propagate across a regular balloon.

### 8.2. Acute Coronary Syndrome

**Figure 18:**
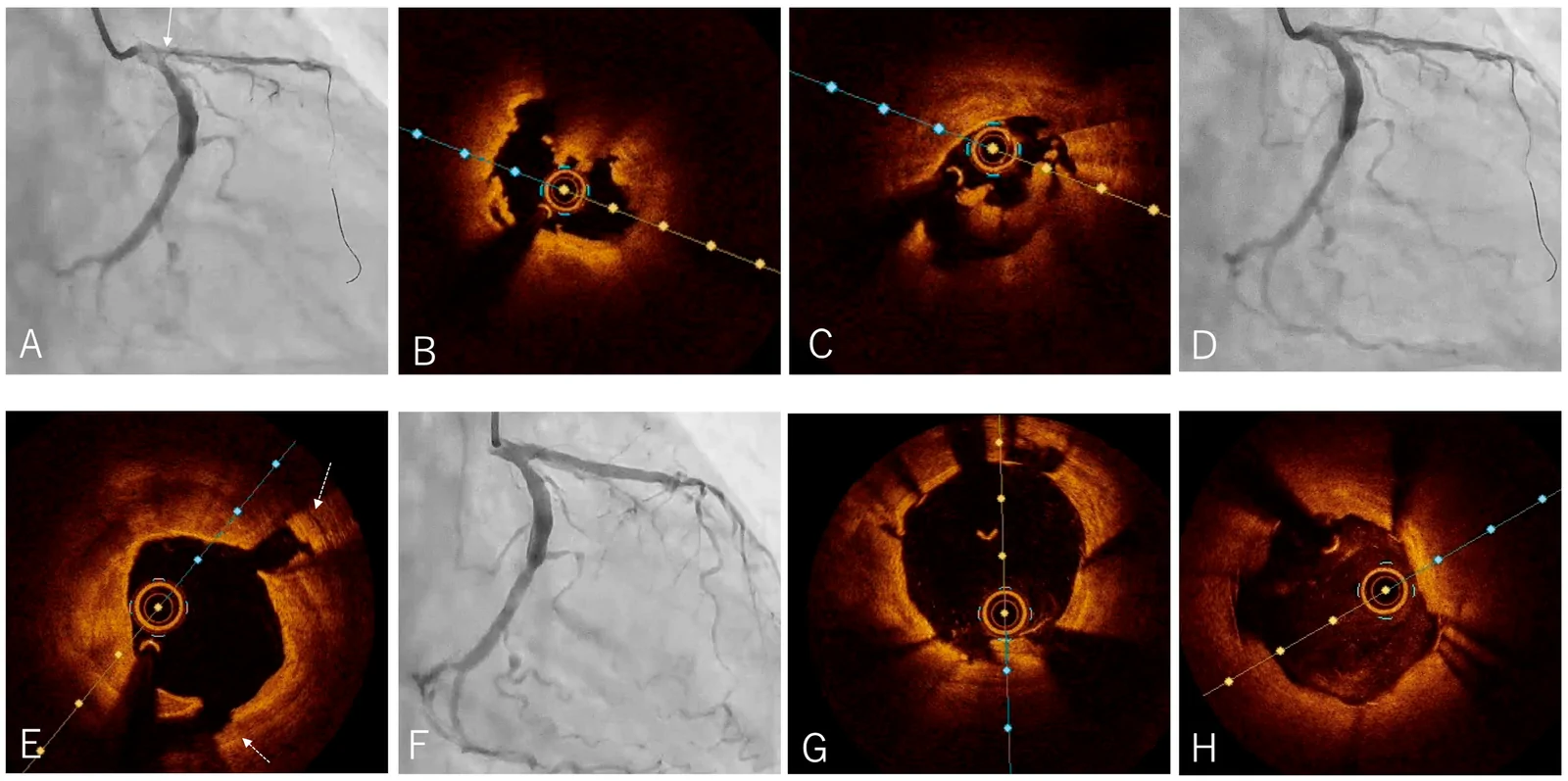
IVL for ST elevation myocardial infarction. (**A**–**C**) Angiogram and OCT after 2.75 mm high-pressure balloon. OCT showed that thrombus was present in the culprit lesion and no fracture was seen in the calcified lesion. (**D**,**E**) Angiogram and OCT after 3.0 mm IVL. OCT showed lumen gain and multiple fractures after 3.0 mm IVL at 2 and 6 o’clock. (**F**–**H**) Angiogram and OCT after DES 3.5 mm. OCT showed adequate dilatation at the culprit and calcified lesion. White arrow indicates the lesion. White dotted arrows indicate the fractures.

The role of IVL during primary PCI for acute coronary syndrome (ACS), especially ST-segment elevation myocardial infarction (STEMI), remains limited. Conventional calcium-modification strategies have important limitations during STEMI, as aggressive balloon dilatation in thrombotic lesions may promote distal embolization, while rotational atherectomy is generally avoided due to no-reflow and increased platelet activation in a highly thrombotic environment. In this setting, IVL represents an emerging alternative that may mitigate some of these risks. Cosgrove et al. reported low rates of procedural complications and in-hospital adverse events with IVL treatment [54]. This favorable safety profile may be related to reduced need for repeated high-pressure balloon inflations. The incidence of MI type 4a was similar to that reported in the REPLICA EPIC 18 study (1.0%) [55] (Figure 18). Although ACS is associated with a relatively lower presence of calcium in the culprit lesion, severe calcification is still found in 10% of ACS patients [56]. Calcification in ACS is associated with high rates of MACE and all-cause mortality at 1 year [57,58,59]. IVL treatment for heavily calcified ACS demonstrates comparable technical success and clinical outcomes when compared to chronic coronary syndrome (CCS), reinforcing its role as an effective intervention even in these acute scenarios [60].

## 9. Comparison Between IVL and Debulking Devices

IVL involves the basic interventional balloon delivery system, has a shorter learning curve, uses workhorse wires, and shows lower rates of procedural complications compared to other debulking devices. RA, OA and excimer laser coronary atherectomy (ELCA; Philips, Netherlands) require a specialized training program. In addition, RA and OA require specialized guidewires. In many cases with calcified nodules, the location was eccentric. If the guidewire position is close to the culprit lesions, debulking devices work well. However, if the guidewire position is close to the normal vessel, debulking devices may pose a risk of vessel injury. In these cases, IVL is a good choice to prepare these lesions. In addition, IVL may be a preferred strategy in ACS patients, because RA and OA can induce slow-flow and/or no-reflow phenomenon.

## 10. Dual Preparation with Debulking Devices and IVL

**Figure 19:**
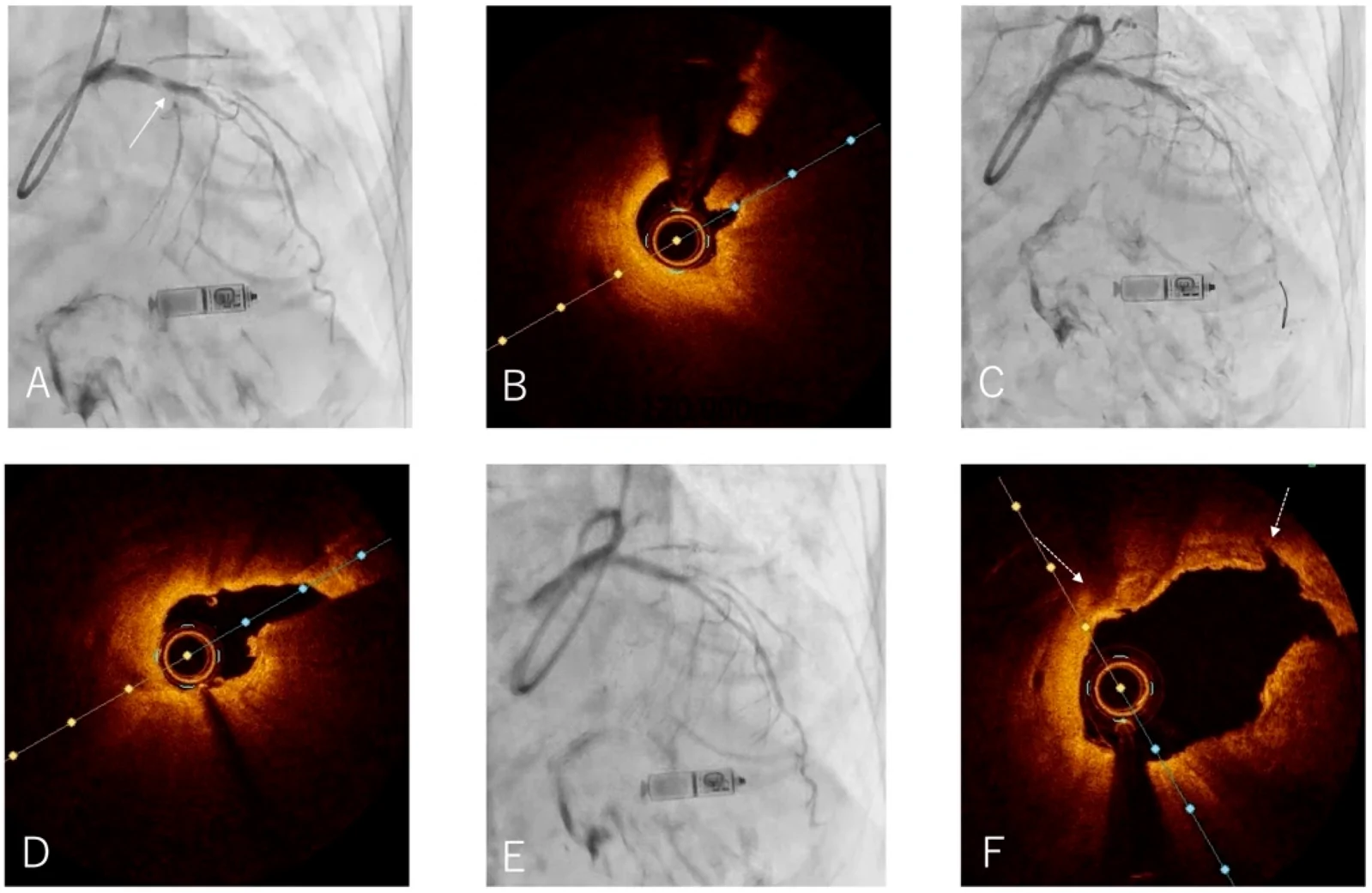
OA followed by IVL for an eruptive calcified nodule. (**A**,**B**) Angiogram and OCT. OCT showed that the lesion was an eruptive calcified nodule. (**C**,**D**) Angiogram and OCT after 80,000 rpm OA. Lumen gain could be obtained. (**E**,**F**) Angiogram and OCT after 3.0 mm IVL. OCT showed that lumen gain and multiple fractures could be seen (2 and 10 o’clock). White arrow indicates the lesion. White dotted arrows indicate the fractures.

**Figure 20:**
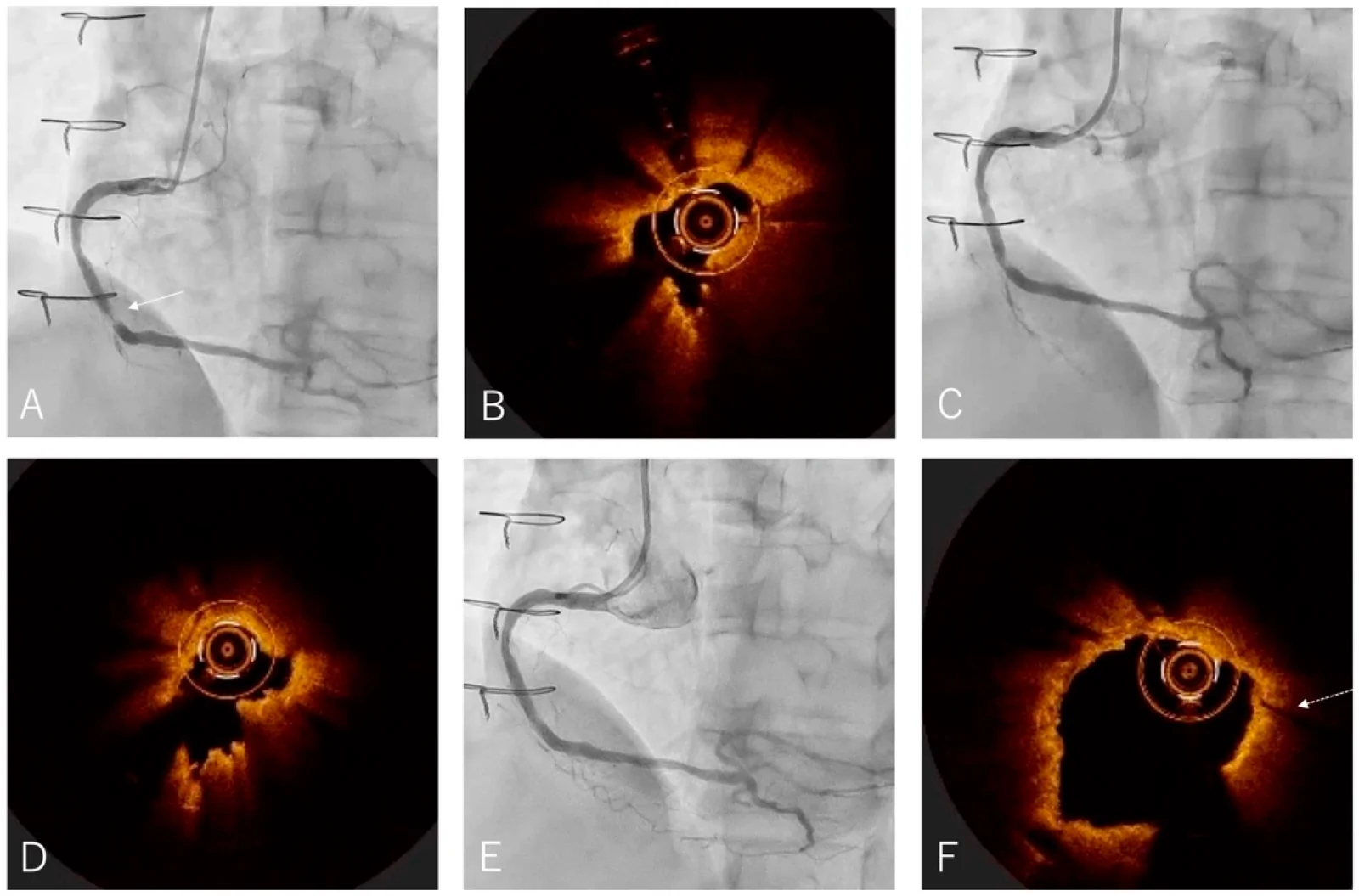
ISR-related calcified nodule treated by ELCA followed by IVL. (**A**,**B**) Angiogram and OCT. OCT showed an ISR-related calcified nodule. (**C**,**D**) Angiogram and OCT after 0.9 mm ELCA (60 mJ/40 Hz). OCT showed vessel modification, and plaque reduction could be seen. (**E**,**F**) Angiogram and OCT after 3.5 mm IVL. OCT showed lumen gain and fracture at 3 o’clock. White arrow indicates the lesion. White dotted arrow indicates the fracture.

The advent of combining IVL with other techniques, such as RA, OA, and ELCA, in challenging lesions has demonstrated enhanced outcomes, suggesting a synergistic potential that warrants further exploration [61,62]. In severely calcified lesions where operators are unable to cross the lesion with the IVL balloons or any balloons, RA and/or OA could be used to facilitate IVL use [63]. In cases with guidewire bias calcified lesions, debulking devices are used to modify the calcified lesions. If the effects of modification were not enough, additional IVL can work well to make fractures and lumen gain (Figure 19). Thrombus reduction and anti-platelet effects of ELCA followed by IVL may be safe and effective for the treatment of eruptive calcified nodules (Figure 20).

## 11. Complications

### 11.1. Vessel Complications

**Figure 21:**
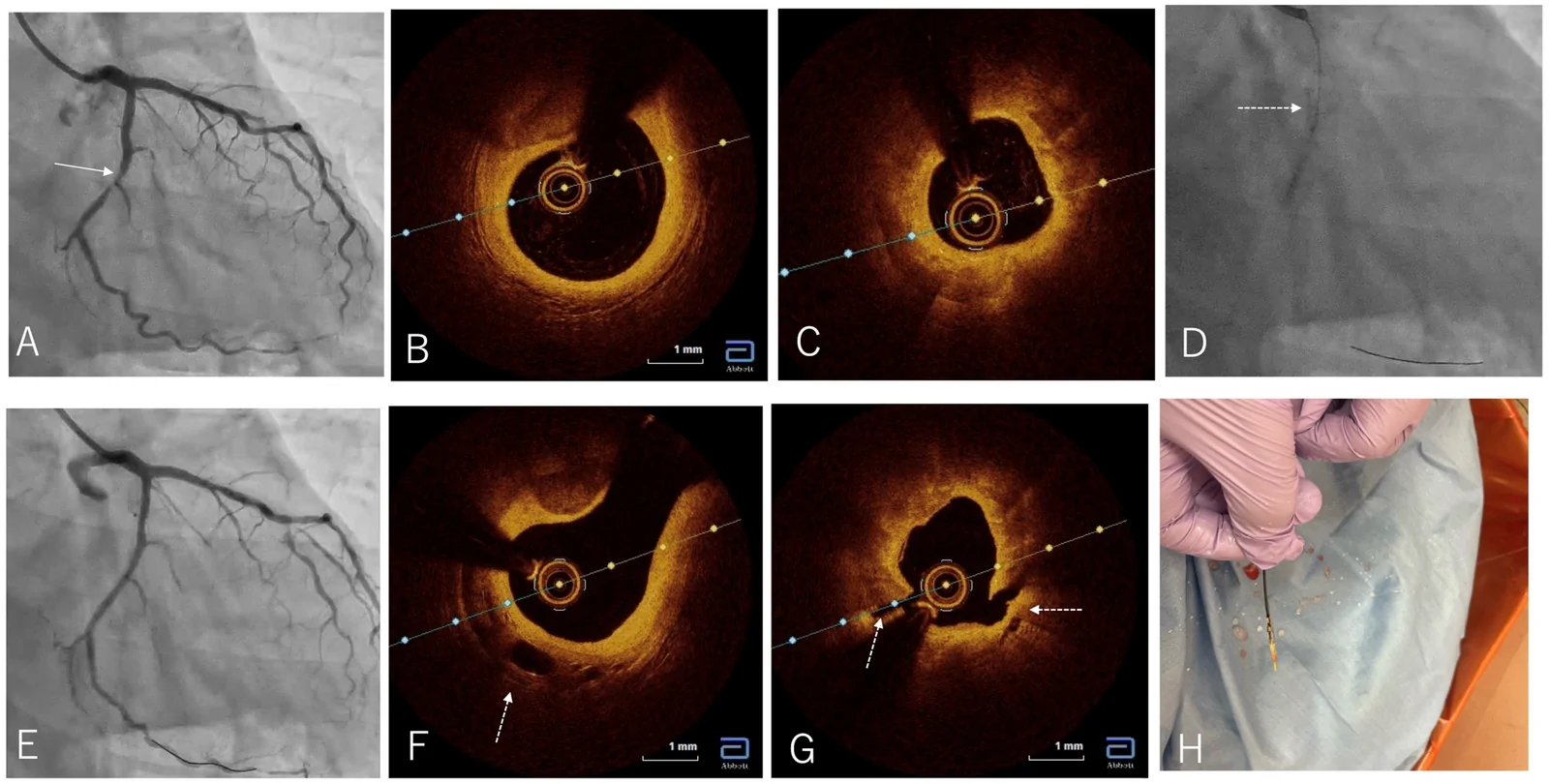
IVL balloon rupture. (**A**–**C**) Angiogram and OCT. (**D**) Angiogram demonstrating contrast leakage due to IVL balloon rupture. (**E**–**G**) OCT showed the occurrence of hematoma and multiple fractures. (**H**) IVL balloon was due to pinhole rupture. White arrow indicates the lesion. White dotted arrows indicate the hematoma and multiple fractures.

Unlike the traditional balloon-based vessel preparation of high-static barotraumatic pressure, IVL generates acoustic mechanical energy with a low-pressure inflated balloon, reducing the possibility of vascular injury. IVL balloon ruptures are due to luminally protruding nodular calcium, significant tortuosity of the coronary artery, and possible gaps in contact between the balloon and the luminal wall. About 13% of IVL balloon ruptures occurred during clinical use [64] (Figure 21). However, unlike balloon rupture with high-pressure balloon dilatation, low-pressure balloon rupture during 4–6 atm with IVL leads to fewer vessel complications. Although coronary perforation after IVL has been reported, IVL leads to vessel perforations at a lower rate because IVL acoustic shockwave penetrates deeply inside the soft tissue with minimal damage and adventitial fibrosis [65]. In Disrupt III, severe dissection occurred in a few cases; however, no perforation occurred after IVL treatment [66].

### 11.2. Slow-Flow or No-Reflow Phenomenon

Slow-flow or no-reflow was never observed in the Disrupt CAD series [11], and we observed a few cases of slow-flow when lipid-rich plaques existed near calcified lesions.

### 11.3. IVL-Induced Arrhythmias

The application of IVL may induce arrhythmia. The acoustic pressure waves produced by IVL are transmitted at a speed of 1 Hz. They can produce a rapidly decaying mechanical energy of 8–10 uJ without the presence of electrical stimulation in the surrounding tissues. When acoustic pressure waves activate stretch-activated ion channels in the cardiac conduction system and depolarize myocardial tissue, the mechanical-electrical coupling process of the heart is completed. Wilson et al. showed that the incidence of coronary IVL-provoked ventricular capture was 77.8%. Ventricular capture was associated with a fall in systolic BP of between 10 and 35 mmHg that immediately resolved on return of intrinsic rhythm. They showed that patients with a heart rate < 65 bpm prior to IVL were over 16 times more likely to experience arrhythmic beats. No adverse clinical events including atrial and ventricular tachyarrhythmias occurred as a result of coronary-IVL-induced capture [67].

## 12. Future Directions

**Figure 22:**
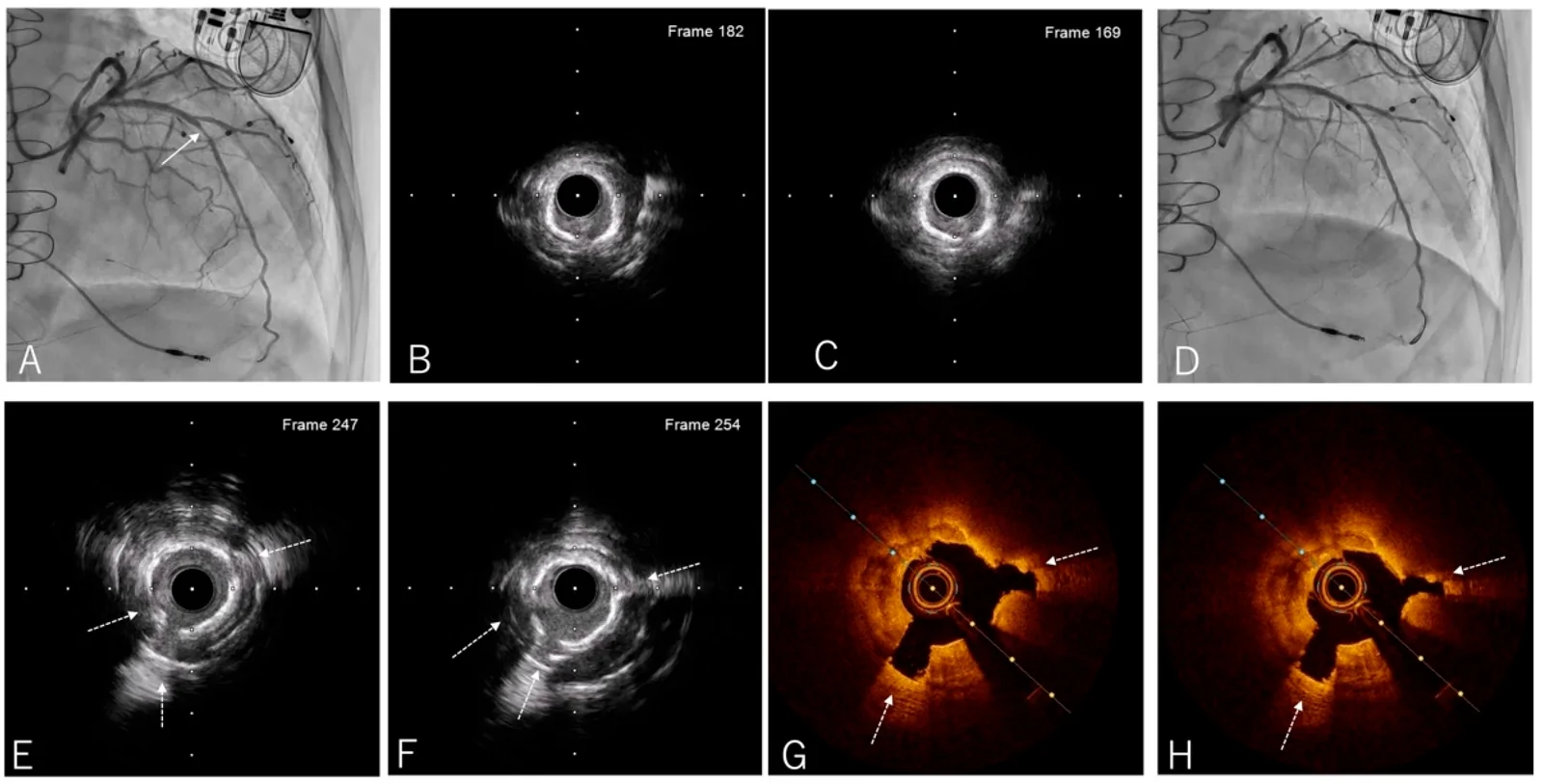
Vessel compliance confirmed by IVUS and OCT stationary methods. (**A**) Angiogram. (**B**,**C**) IVUS in systolic and diastolic phases. (**D**) Angiogram after 2.5 mm IVL. (**E**,**F**) IVUS in systolic and diastolic phases. Increased vessel compliance and multiple fractures can be seen at 3, 7, and 8 o’clock. (**G**,**H**) OCT in systolic and diastolic phases. Increased vessel compliance and multiple fractures could be seen at 3 and 7 o’clock. Wider fractures could be seen in the systolic phase compared to the diastolic phase. White arrow indicates the lesion. White dotted arrows indicate multiple fractures.

**Figure 23:**
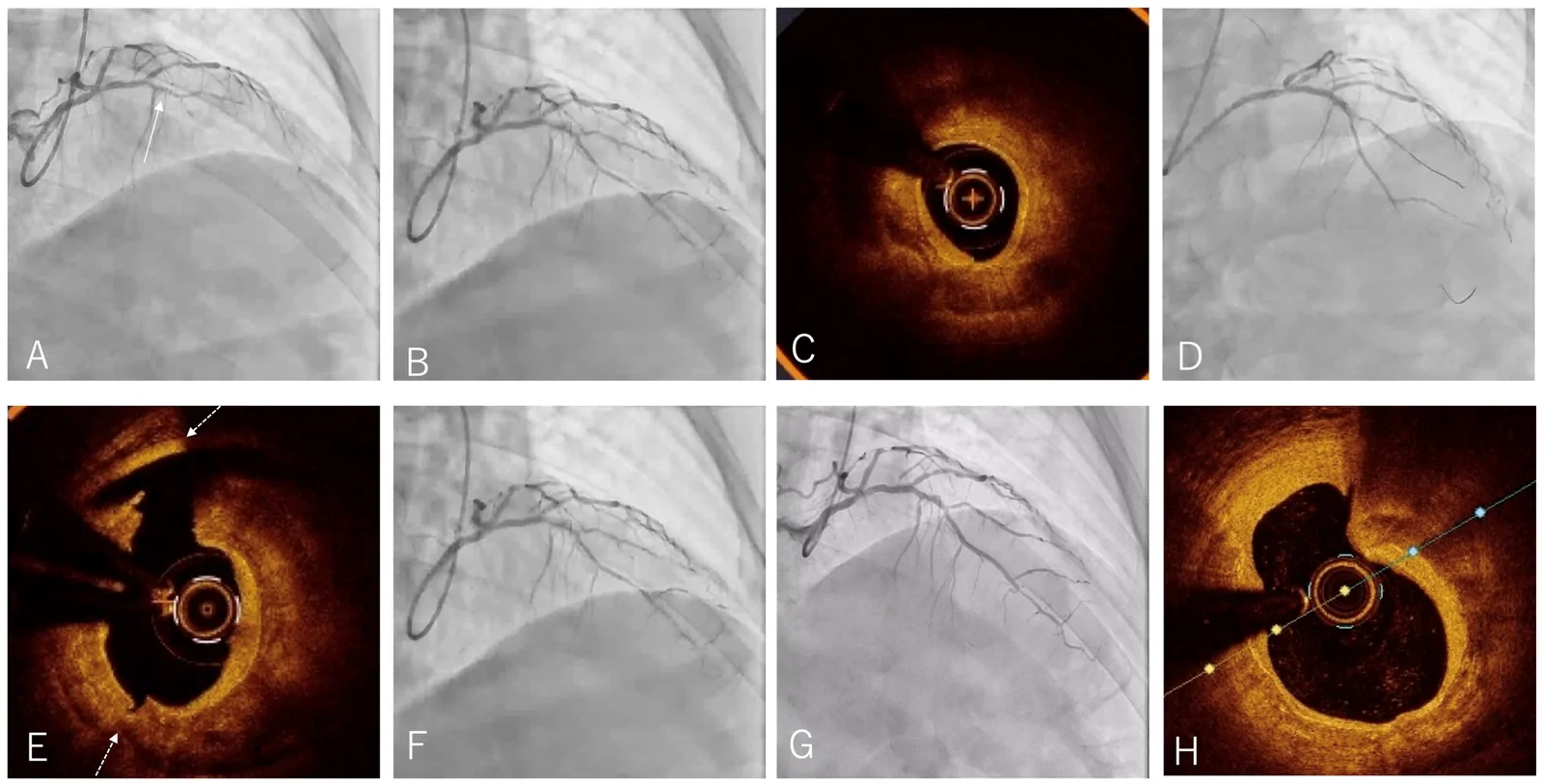
IVL followed by DCB strategy. (**A**) Angiogram showed a CTO lesion. (**B**,**C**) Angiogram and OCT after 2.5 mm high-pressure balloon dilatation. OCT showed a superficial calcified sheet without fracture. (**D**,**E**) Angiogram after 2.5 mm IVL. OCT showed lumen gain and multiple fractures could be seen at 12 and 7 o’clock. (**F**) Final angiogram after 2.5 mm DCB treatment. (**G**,**H**) Angiogram and OCT at the 12-month follow-up. Angiogram was acceptable and OCT showed late lumen enlargement. White arrow indicates the lesion. White dotted arrows indicate the fractures.

**Figure 24:**
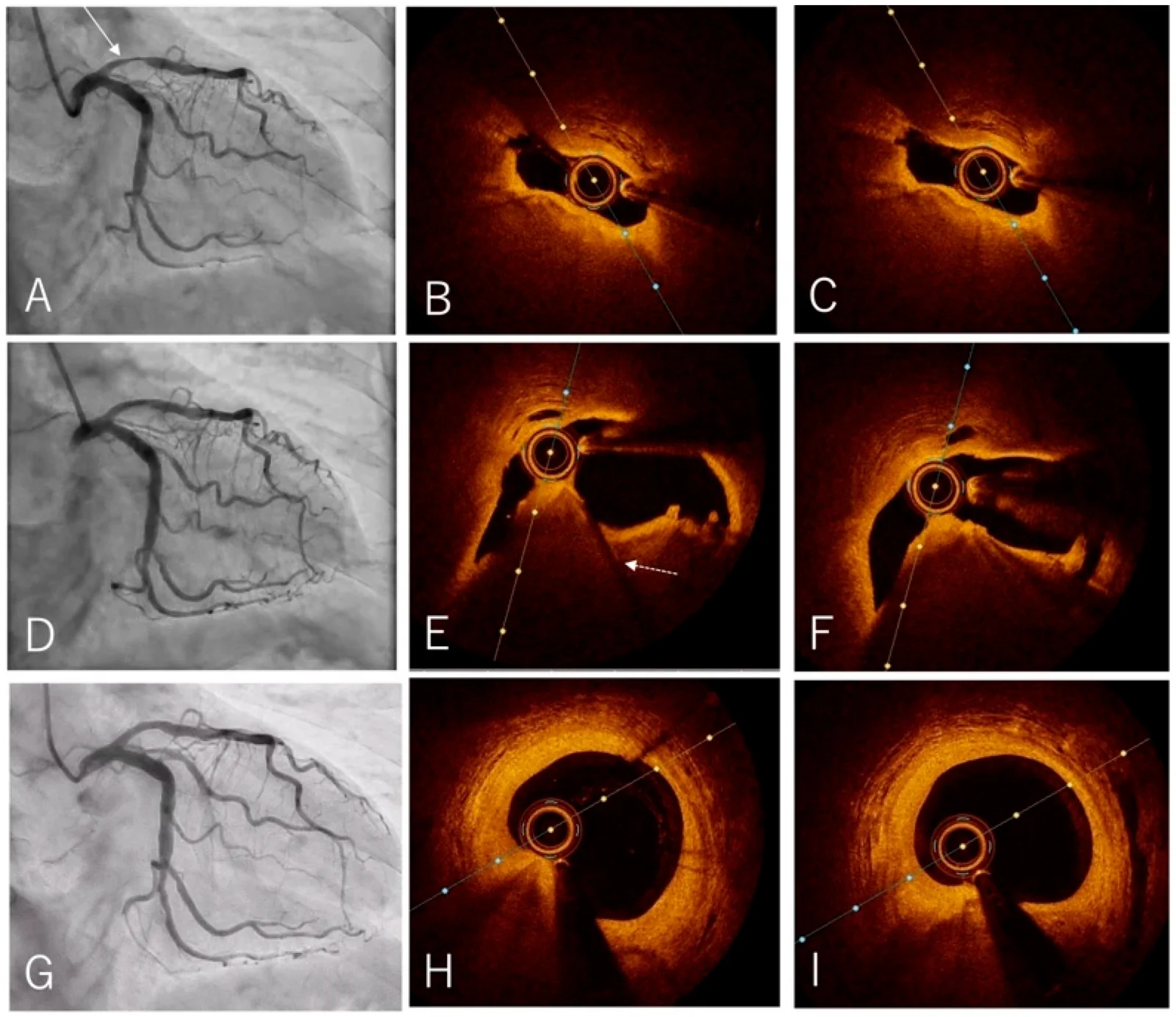
IVL followed by DCB strategy for eccentric calcium. (**A**) Angiogram. (**B**,**C**) OCT in systolic and diastolic phases. Vessel compliance was low. (**D**) Angiogram followed by 3.0 mm IVL. (**E**,**F**) OCT in systolic and diastolic phases. Increased vessel compliance was observed, and fractures could be seen at the 5 o’clock position. (**G**) Angiogram at the 12-month follow-up. (**H**,**I**) OCT in systolic and diastolic phase. Increased vessel compliance and late lumen gain could be seen. White arrow indicates the lesion. White dotted arrow indicates the fracture.

Coronary artery compliance can be measured by IVUS by assessing dynamic changes in the vessel lumen during systole and diastole [68,69]. Oliveri et al. demonstrated that IVL leads to increased vessel compliance and ensures stent expansion. We have found that IVL leads to an increase in vessel compliance by using both IVUS and OCT stationary methods, as illustrated in Figure 22. Oliveri et al. also suggested that multiple macrofractures and microfractures induced by IVL provide two key advantages. One is improved vessel compliance, reducing the need for high radial forces to maintain luminal expansion. Second is the enhanced drug diffusion, allowing for more effective drug penetration into the vessel wall [70]. Since DES may interfere with the vessel’s natural remodeling capacity, an IVL followed by a DCB strategy may be an effective strategy if increased vessel compliance is maintained for long-term periods [71,72] (Figure 23 and Figure 24).

## 13. A New Algorithm for Coronary Calcification

**Figure 25:**
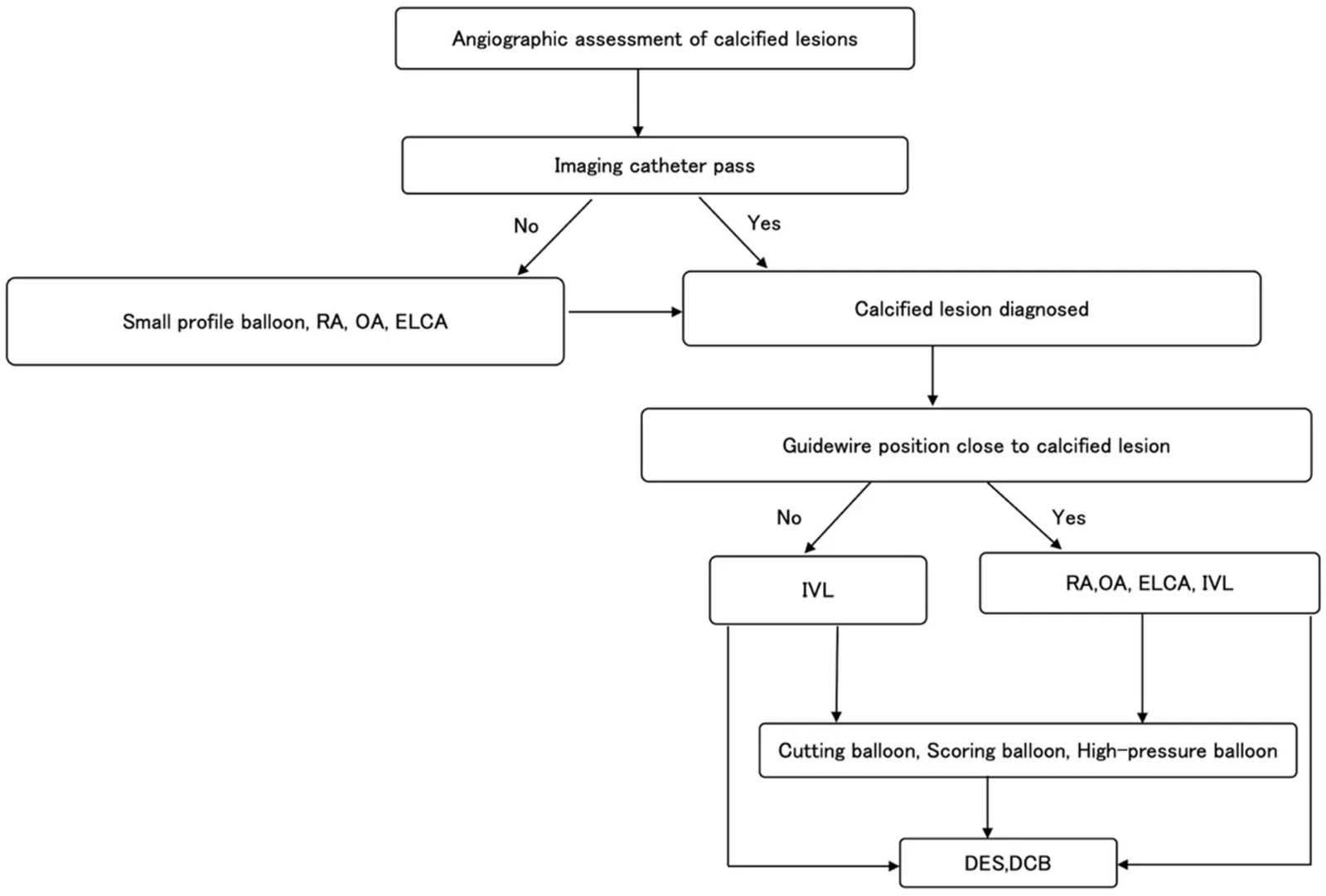
A new algorithm for coronary calcification treatment..

The algorithm begins with assessing coronary calcified lesions using intravascular imaging (Figure 25). The decision regarding which modality of calcium modification to use should be guided by the findings of intracoronary imaging, lesion characteristics, device availability and operator’s preference. Because RA, OA and ELCA work through guidewire bias, guidewire position should be analyzed. RA is preferred when a calcified lesion is located near the guidewire. OA is preferred for larger caliber arteries and aorto-ostial lesions. ELCA is preferred for thrombus-containing lesions. If the guidewire position is located near non-calcified lesions, there is a risk of complication such as dissection. IVL is expected to become a predominant treatment modality due to its ease of use and lower risk of major complications.

## 14. Conclusions

The efficacy in fragmenting calcium deposits through pulsatile sonic waves supports safer and more effective vessel preparation for stent deployment and DCB strategy.

## Figures and Tables

**Table 1:** Comparison of coronary artery modification devices.

Parameter	Intravascular Lithotripsy	Rotational Atherectomy	Orbital Atherectomy	Excimer Laser Coronary Atherectomy
Guidewire	Workhorse wire	0.009–0.014 tip Rota wire	0.012–0.014 inch Viper wire	Workhorse wire
Guidewire bias	No	Yes	Yes	Yes
Sonic wave effect	Yes	No	No	Yes
Plaque reduction	No	Yes	Yes	Yes

## Data Availability

No new data were created or analyzed in this study. Data sharing is not applicable to this article.

## Abbreviations

The following abbreviations are used in this manuscript.

RA	Rotational atherectomy
OA	Orbital atherectomy
ELCA	Excimer laser coronary atherectomy
IVL	Intravascular lithotripsy
DCB	Drug-coated balloon
PCI	Percutaneous coronary intervention
CAD	Coronary artery disease
MACE	Major adverse cardiac events
OCT	Optical coherence tomography
IVUS	Intravascular ultrasound
GEC	Guide extension catheter
DES	Drug-eluting stent
ISR	In-stent restenosis
CN	Calcified nodule
SB	Side branch
MB	Main branch
LAD	Left anterior descending artery
ACS	Acute coronary syndrome
STEMI	ST-segment elevation myocardial infarction
